# Sulfur‐Free Radical RAFT Polymerization of Methacrylates in Homogeneous Solution: Design of *exo*‐Olefin Chain‐Transfer Agents (R−CH_2_C(=CH_2_)Z)

**DOI:** 10.1002/anie.202212633

**Published:** 2022-11-23

**Authors:** Maki Amano, Mineto Uchiyama, Kotaro Satoh, Masami Kamigaito

**Affiliations:** ^1^ Department of Molecular and Macromolecular Chemistry Graduate School of Engineering Nagoya University Furo-cho, Chikusa-ku Nagoya 464-8603 Japan; ^2^ Department of Chemical Science and Engineering School of Materials and Chemical Technology Tokyo Institute of Technology 2-12-1-H120 Ookayama Meguro-ku Tokyo 152-8550 Japan

**Keywords:** Block Polymer, Living Polymerization, Methacrylate, RAFT Polymerization, Radical Polymerization

## Abstract

In this work, the development of *exo*‐olefin compounds (R−CH_2_C(=CH_2_)Z) as chain‐transfer agents for the sulfur‐free reversible addition‐fragmentation chain transfer (RAFT) radical polymerization of methacrylates in homogeneous solution is described. A series of *exo*‐olefin compounds with a methyl methacrylate (MMA) dimer structure as the R group and a substituted α‐methylstyrene unit as the −CH_2_C(=CH_2_)Z (Z: Ph−Y) group were synthesized and used for the radical polymerization of MMA in toluene and PhC(CF_3_)_2_OH. These compounds underwent transfer of the CH_2_C(=CH_2_)Z group via addition‐fragmentation of the propagating methacryloyl radical. More electron‐donating (Y) substituents, such as methoxy and dimethylamino groups, produced polymers with narrower molecular weight distributions. A continuous monomer addition method further improved molecular weight control and enabled the synthesis of colorless, sulfur‐free, multiblock copolymers of methacrylates in homogeneous solutions.

## Graphical abstract

This work develops *exo*‐olefin compounds (R−CH_2_C(=CH_2_)Z) as chain‐transfer agents for the sulfur‐free RAFT polymerization of methacrylates in homogeneous solution. A series of R−CH_2_C(=CH_2_)Z with the MMA‐dimer structure as R and substituted α‐methylstyrene unit as −CH_2_C(=CH_2_)Z (Z: Ph−Y) were synthesized. More electron‐donating substituents (Y) work better. Colorless sulfur‐free multiblock copolymers were prepared via sequential monomer additions.

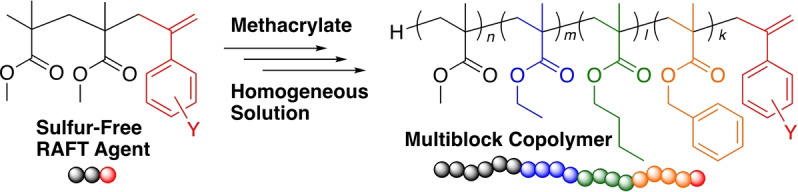

## Introduction

**Scheme 1:**
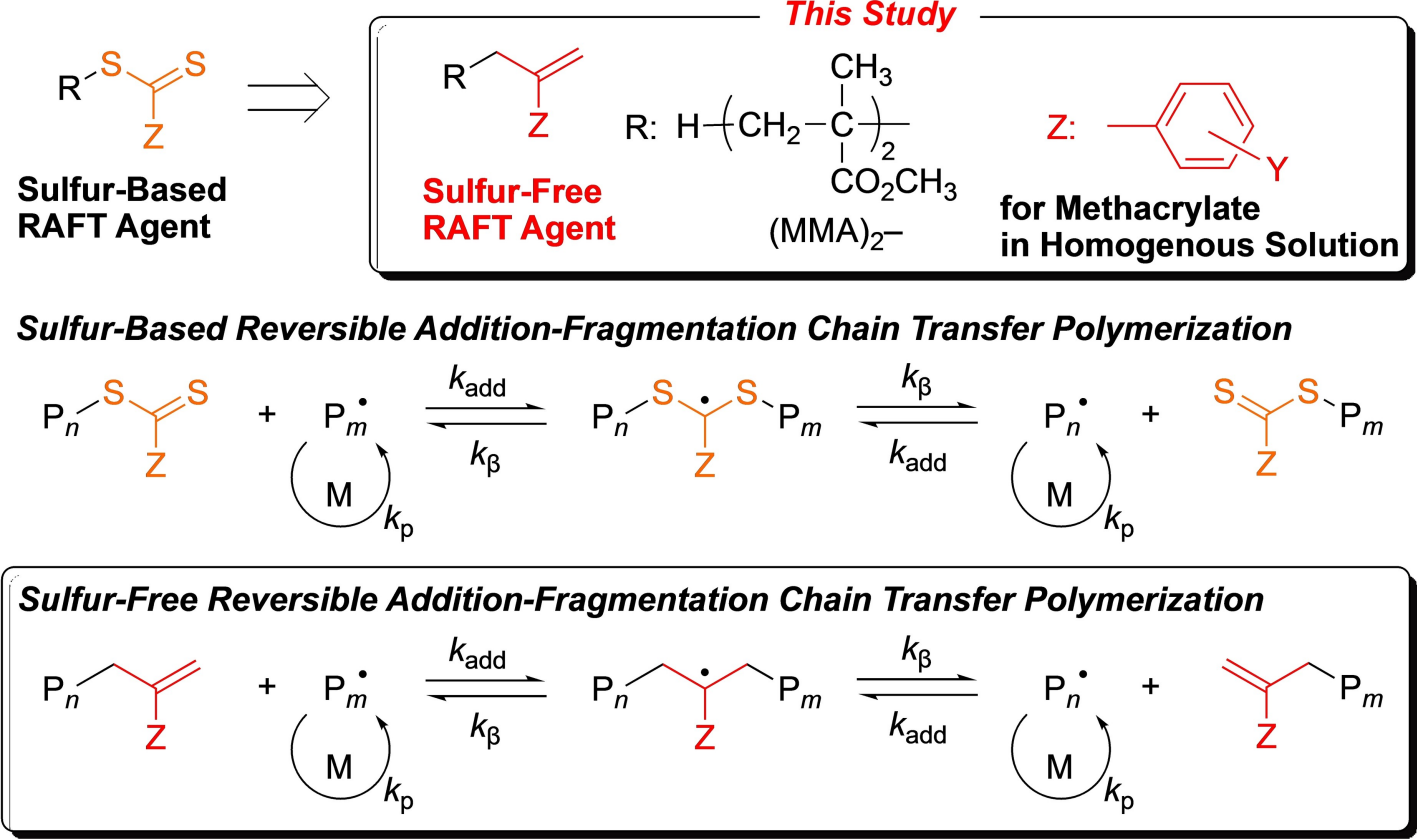
Sulfur‐based and sulfur‐free reversible addition‐fragmentation chain transfer polymerization.

The addition‐fragmentation reaction is common in not only organic but also polymer chemistry.[^1^, ^2^, ^3^, ^4^] In particular, developing reversible processes is important for enabling precision polymer synthesis.[^5^, ^6^, ^7^, ^8^, ^9^, ^10^, ^11^, ^12^, ^13^, ^14^, ^15^, ^16^, ^17^, ^18^] Reversible addition‐fragmentation chain transfer polymerization (RAFT polymerization), is recognized as one of the most efficiently controlled or reversible deactivation radical polymerizations (RDRPs). This reaction uses thiocarbonylthio (R−SC(=S)Z) compounds as chain‐transfer agents,^[19]^ which undergo the addition of propagating radical species to the C=S bond followed by β‐scission of the C−S bond (Scheme 1).

However, the term RAFT can be used in a more general sense to describe a reversible chain‐transfer reaction,^[5]^ which consists of the addition of any propagating species to an unsaturated bond originating from the chain‐transfer agent to form an adduct intermediate and its subsequent fragmentation via β‐scission into another propagating species and a dormant polymer terminal with the same unsaturated bond. In this sense, an *exo*‐olefin compound such as R−CH_2_C(=CH_2_)Z can work as a RAFT agent via the addition of propagating radical species to the C=C bond followed by β‐scission of the C−C bond. In this case, the transferable group is not −SC(=S)Z but instead −CH_2_C(=CH_2_)Z and is thus free from sulfur. Such sulfur‐free RAFT agents have long been desired for the preparation of colorless and odorless polymers, particularly in view of their applications. However, the RAFT process for *exo*‐olefin compounds is generally slow, and the chain‐transfer constants of *exo*‐olefin compounds are low in comparison to those of thiocarbonylthio compounds due to less reactive C=C bonds and stronger C−C bonds.^[5]^

Prior to the advent of efficient sulfur‐based RAFT agents,^[19]^ an *exo*‐olefin‐terminated poly(methacrylate) (**P**_***n***_: H−(CH_2_C(CH_3_)(CO_2_R′))_*n*_−CH_2_C(=CH_2_)CO_2_R′), which was prepared by cobalt‐mediated catalytic chain‐transfer polymerization of methacrylate (CH_2_=C(CH_3_)CO_2_R′), was used as a “macromonomer” for the heterogeneous radical emulsion polymerization of another methacrylate (CH_2_=C(CH_3_)CO_2_R′′).^[20]^ Although the *exo*‐olefin‐terminated polymer was named a macromonomer in the literature, it worked as macro chain‐transfer agent, which generates propagating poly(methacrylate) radicals via β‐scission to produce block copolymers with relatively narrow molecular weight distributions (MWDs) (*M*_w_/*M*_n_=1.2–1.4) and controlled molecular weights via the RAFT mechanism. This emulsion polymerization was further used for the synthesis of multiblock polymers with different methacrylates via slow continuous monomer addition.[^21^, ^22^, ^23^, ^24^, ^25^] Although the chain‐transfer constants of the *exo*‐olefin‐terminated polymers are not very high (*C*_tr_ ≈0.2),[^26^, ^27^] the emulsion polymerization that occurs in a confined space, into which the added monomer is slowly supplied from monomer droplets emulsified in the aqueous phase, allows substantially fast chain transfer in comparison to propagation and diminishes termination to enhance molecular weight control. However, sulfur‐free RAFT polymerization in homogeneous solutions is still a major challenge in radical polymerization.

**Scheme 2:**
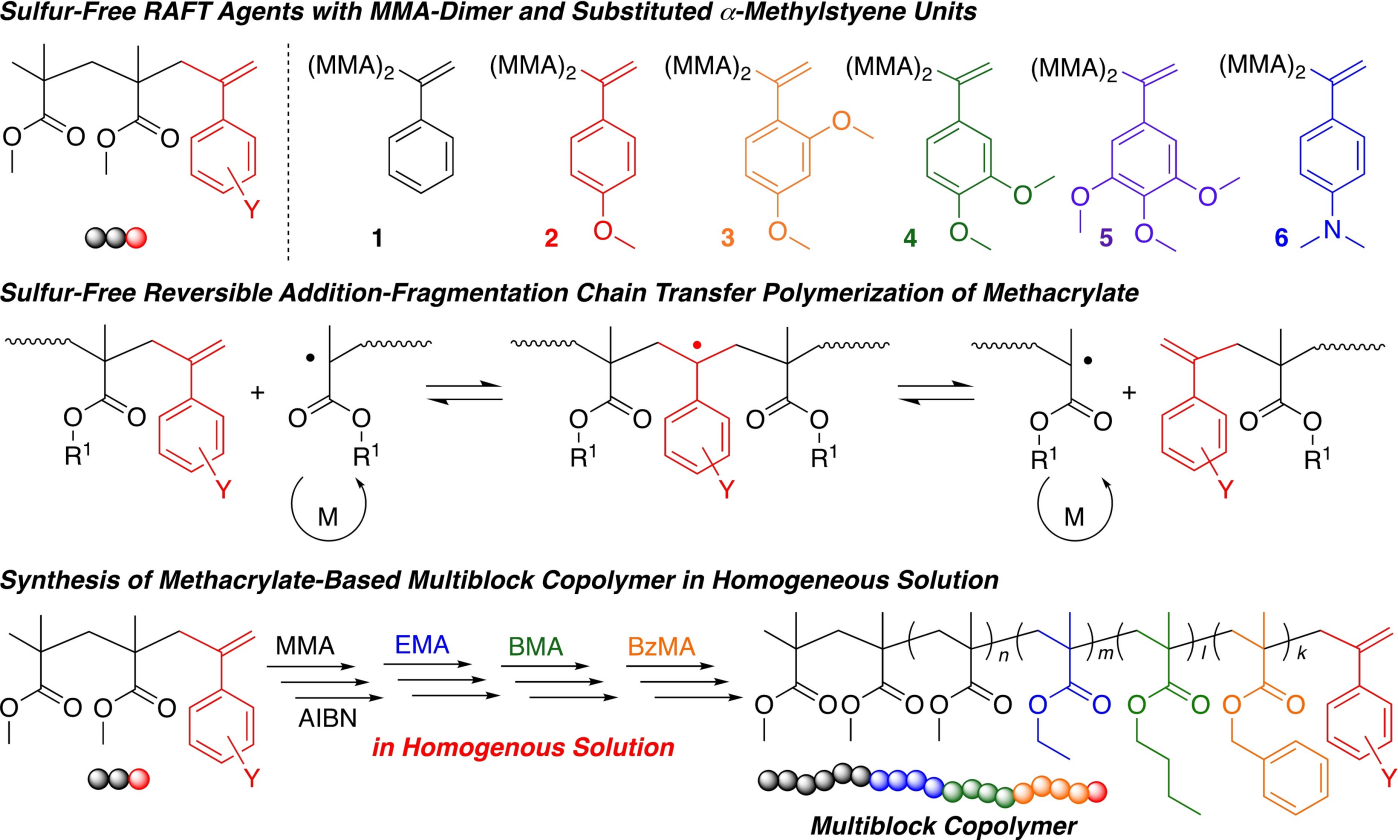
Sulfur‐free RAFT agents with MMA‐dimer and substituted α‐methylstyrene unit for polymerization of methacrylates and multiblock copolymers in homogeneous solution.

This study aimed to develop a new and efficient sulfur‐free *exo*‐olefin‐based RAFT agent by designing appropriate R and Z groups in R−CH_2_C(=CH_2_)Z to control the homogeneous radical polymerization of methacrylates (Scheme 2). For the R group, we chose a dimer of methyl methacrylate (MMA) (H−(CH_2_C(CH_3_)(CO_2_Me))_2_−) because the chain‐transfer constants of a series of *exo*‐olefin‐terminated poly(MMA) (PMMA) molecules (**P**_***n***_, R′=Me) are almost the same (*C*_tr_ ≈0.2) when *n*≥2 in **P**_***n***_ due to similar steric hindrance around the cleavable C−C bond.^[26]^ On the other hand, for the Z group, we employed a series of aryl groups (Ph−Y) that possess particularly electron‐donating substituents (Y) because an electron‐rich C=C bond should have relatively high reactivity toward nucleophilic methacryloyl radicals to facilitate the addition reaction in the RAFT process. The −CH_2_C(=CH_2_)Ph−Y moiety can be regarded as an α‐methylstyrene unit, which should have higher reactivity toward the methacryloyl radical than the −CH_2_C(=CH_2_)CO_2_Me moiety in **P**_***n***_. The reactivity of the *exo*‐olefin‐based RAFT agent can consequently be enhanced with an electron‐donating substituent analogous to the copolymerizability of MMA and substituted styrenes.^[28]^ Although α‐methylstyrene dimers and mixtures of α‐methylstyrene and methacrylate heterodimers have been prepared and used as chain‐transfer agents, they are not as efficient as **P**_***n***_.[^29^, ^30^, ^31^, ^32^] Here, we synthesized a series of MMA dimers with substituted α‐methylstyrene units (H−(CH_2_C(CH_3_)(CO_2_Me))_2_−CH_2_C(=CH_2_)Ph−Y) and used them as chain‐transfer agents for the RAFT polymerization of MMA and block copolymer syntheses in homogeneous solutions.

## Results and Discussion

**Scheme 3:**
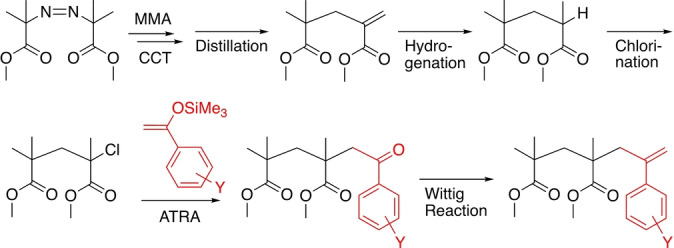
Synthesis of sulfur‐free RAFT agents with MMA‐dimer and substituted α‐methylstyrene unit via catalytic chain‐transfer (CCT) oligomerization of MMA, hydrogenation, chlorination, atom transfer radical addition (ATRA) of silyl enol ether, and Wittig reaction.

For the synthesis of the *exo*‐olefin‐based chain‐transfer agents, several reactions related to controlled radical additions and polymerizations were utilized (Scheme 3). An MMA dimer olefin (**P_1_**: CH_3_C(CH_3_)(CO_2_Me)−CH_2_C(=CH_2_)CO_2_Me) was first prepared by the cobalt‐mediated catalytic chain‐transfer (CCT) oligomerization of MMA^[33]^ and purified by distillation. Then, **P_1_** was hydrogenated and chlorinated for conversion into an MMA dimer chloride (H−(CH_2_C(CH_3_)(CO_2_Me))_2_−Cl), which can also work as an efficient initiator for the metal‐catalyzed living radical polymerization of MMA.^[34]^

Kharasch or atom transfer radical addition (ATRA) of the MMA dimer chloride and a series of unsubstituted and substituted benzaldehyde silyl enol ethers (4‐methoxy, 2,4‐dimethoxy, 3,4‐dimethoxy, 3,4,5‐trimethoxy, and 4‐dimethylamino) was conducted using CuCl and 2,2′‐bipyridine as catalysts. This reaction was similar to the previously reported end‐functionalization of PMMA chloride with benzaldehyde silyl enol ether, which resulted in a benzophenone terminus during the ruthenium‐catalyzed living radical polymerization of MMA.[^35^, ^36^] Even for the MMA dimer chloride and various silyl enol ethers using copper catalysts, radical addition and subsequent desilylation efficiently occurred, resulting in an MMA dimer with substituted benzophenone groups.

**Figure 1:**
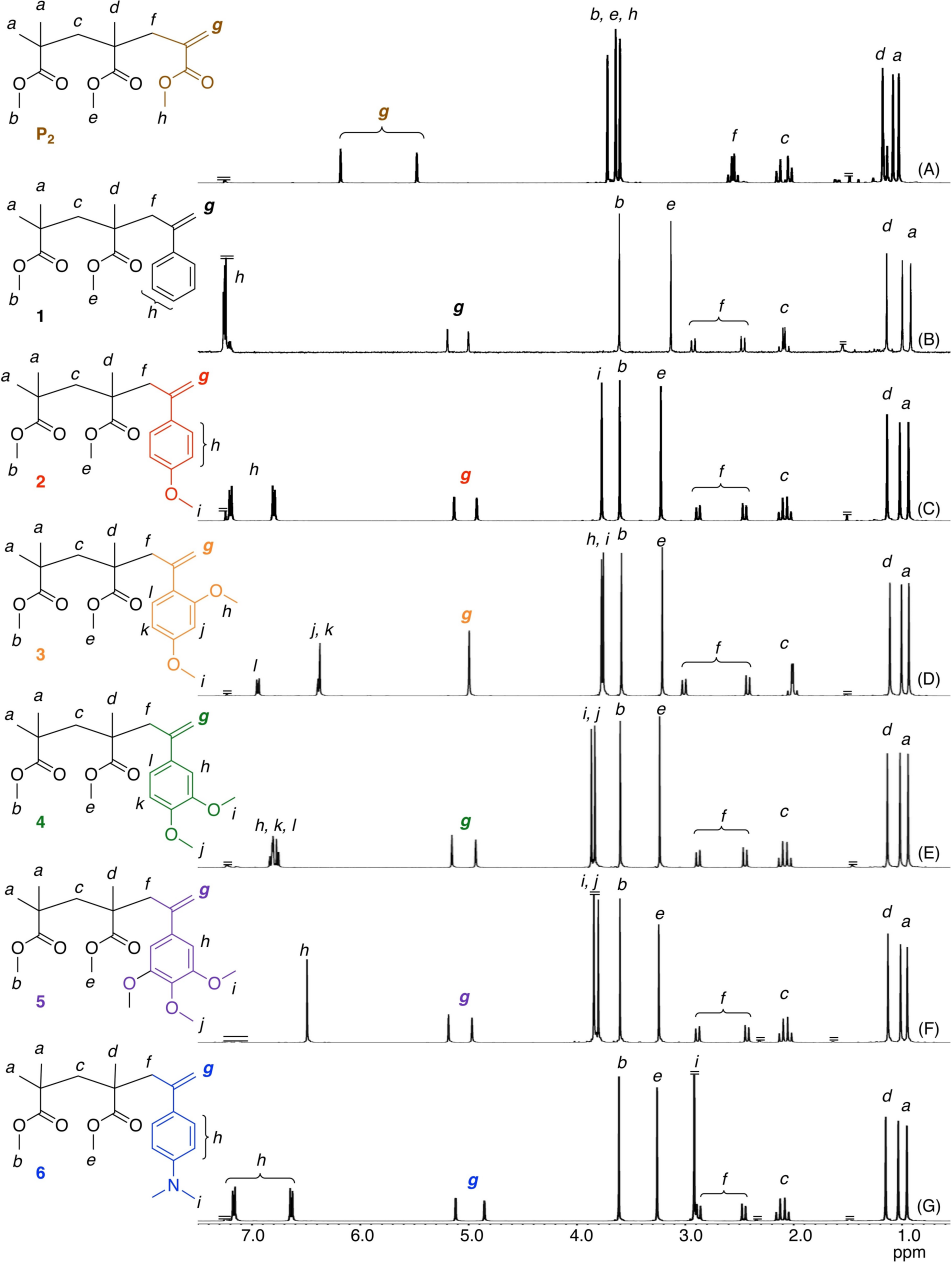
^1^H NMR spectra (CDCl_3_, 55 °C) of sulfur‐free RAFT agents (**P**_2_ (A), **1** (B), **2** (C), **3** (D), **4** (E), **5** (F) **6** (G)).

The ketone groups were then transformed into *exo*‐olefins using phosphorous ylide (Wittig reagent) or Tebbe reagent, where the latter was used for only bulky ketones with 2,4‐dimethoxy or 3,4,5‐trimethoxy groups, resulting in a series of MMA dimers with substituted α‐methylstyrene units (**1**–**6**). All peaks in the ^1^H (Figure 1) and ^13^C NMR (Figures S1 and S2) spectra were assigned to the corresponding protons and carbons of the series of target compounds, indicating their successful preparation. In particular, the methylene protons and carbons of **1**–**6** were all observed at significantly high magnetic fields in comparison to those of the *exo*‐olefin‐ended MMA trimers **P_2_**, which were prepared by the cobalt‐mediated catalytic chain‐transfer oligomerization of MMA followed by purification as described previously,^[26]^ suggesting that their *exo*‐olefins are at least electron rich. However, detailed discussions on the effects of the substituents on the chemical shifts and electron densities of the C=C bonds are challenging.

The homogeneous radical polymerization of MMA was investigated using 2,2′‐azobisisobutyronitrile (AIBN) in the absence and presence of **2** or **P_2_** in toluene at 60 °C. In the absence of any chain‐transfer agents, high molecular weight polymers (*M*_n_=182 300) were produced in 24 h with 92 % monomer conversion (Figures S3A–S5A). In the presence of **P_2_** or **2**, the polymerization became slightly slower and resulted in polymers with relatively low molecular weights. More specifically, the *M*_n_ value obtained with **2** was lower than that obtained with **P_2_** (*M*_n_=16 700 vs. 20 500). Furthermore, the *M*_n_ values decreased along with monomer conversion and approached the calculated values assuming that one molecule of **2** generates one polymer chain. These results indicate that **2** is superior to **P_2_**, which is among the most effective sulfur‐free RAFT agents in the radical polymerization of MMA.^[26]^ However, the final *M*_n_ value was still higher than the calculated value, indicating that all molecules of **2** did not participate in the polymerization.

The solvent was then changed to a fluoroalcohol, PhC(CF_3_)_2_OH, which has been reported to increase the addition rate of the PMMA radical to the styrenic moiety of **2** via the hydrogen bonding of the acidic alcohol to the carbonyl group at the chain‐end of the PMMA radical because of an enhanced effect of PhC(CF_3_)_2_OH on copolymerization of (meth)acrylic monomers and styrenic or olefinic monomers.[^37^, ^38^, ^39^, ^40^, ^41^, ^42^] The polymerization in PhC(CF_3_)_2_OH without using any chain transfer agents was slightly faster and resulted in higher molecular weight polymers (*M*_n_=274 300) (Figures S3B–S5B). Upon the addition of **P_2_** or **2**, the molecular weights were significantly lowered. In particular, the final *M*_n_ obtained with **2** became even lower (*M*_n_=14 300) than that with **2** in toluene (*M*_n_=16 700). These results indicate that fluoroalcohol is a suitable solvent for enhancing the chain‐transfer ability of **2** to generate PMMA radicals.

**Figure 2:**
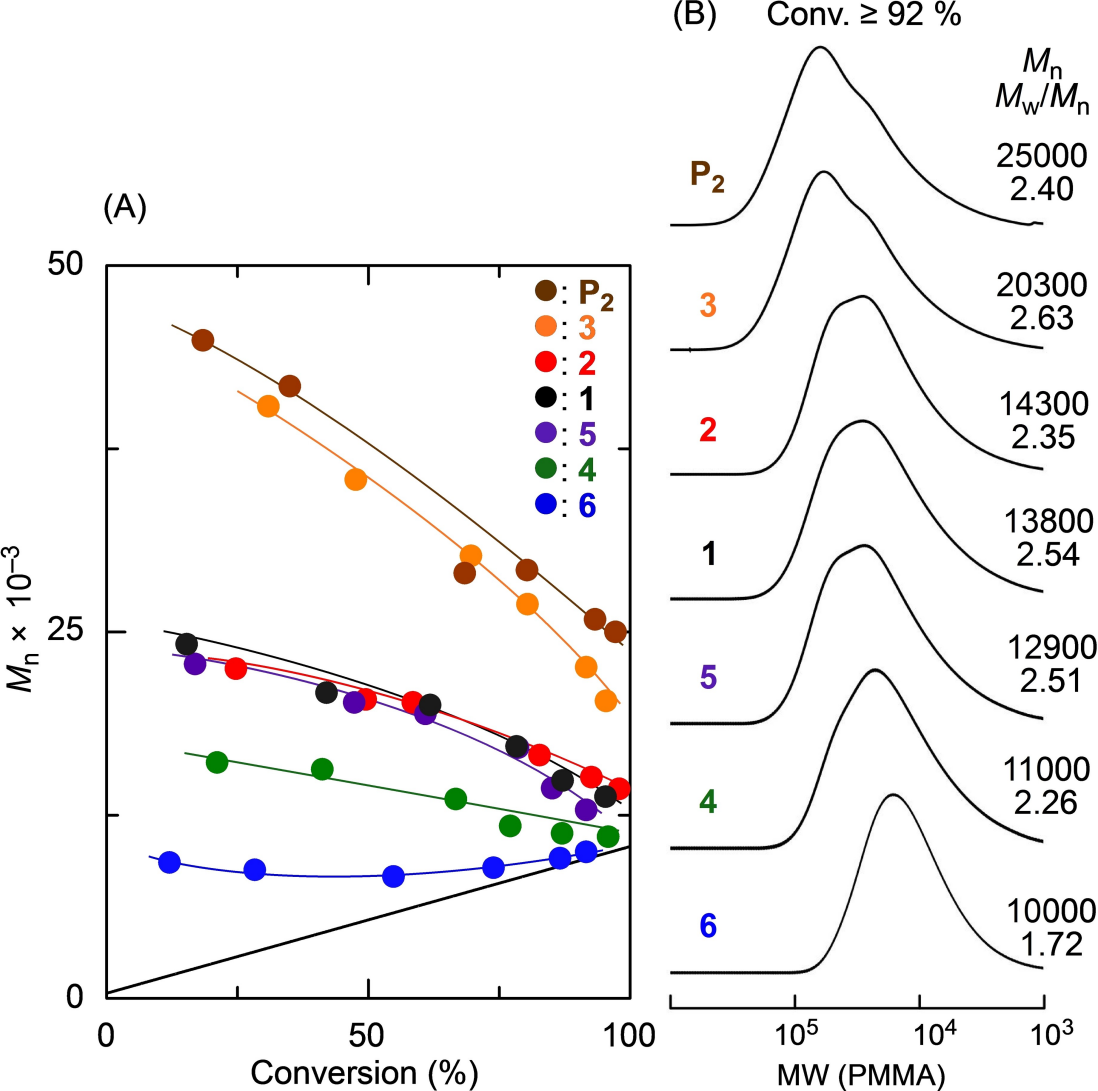
*M*_n_ values (A) and SEC curves (B) of PMMA obtained in radical polymerization of MMA in the presence of various sulfur‐free RAFT agents in PhC(CF_3_)_2_OH at 60 °C: [MMA]_0_/[RAFT agent]_0_/[AIBN]_0_=5000/50/5.0 mM.

The series of *exo*‐olefin‐based chain‐transfer agents (**1**–**6**) were then evaluated in the radical polymerization of MMA with AIBN in PhC(CF_3_)_2_OH at 60 °C. All polymerizations proceeded almost quantitatively in 60 h, although the rates changed slightly with the structures (Figure S6). The molecular weights of the polymers depended significantly on the substituents and decreased in the following order: **3**>**1**≈**2**≈**5**>**4**>**6** (Figure 2). In particular, the *M*_n_ values obtained with **4** and **6** were consistently lower than those obtained with **2** and finally became very close to the calculated value (*M*_n_=10 300), indicating that almost all **4** and **6** molecules worked as chain‐transfer agents under homogeneous conditions. Therefore, MMA dimers with 3,4‐dimethoxy and 4‐dimethylamino styrene units are more effective sulfur‐free chain‐transfer agents for radical polymerization of MMA than the previously reported *exo*‐olefin‐terminated PMMA (**P**_***n***_). However, those with bulky substituents, such as 2,4‐dimethoxy and 3,4,5‐trimethoxy styrene units, were less efficient. Similar tendencies were observed in toluene (Figures S7 and S8), although the obtained molecular weights were slightly higher than those obtained in PhC(CF_3_)_2_OH.

Among various methods to calculate chain‐transfer constants (*C*_tr_),[^43^, ^44^, ^45^] the method based on an equation for the *M*_n_‐conversion plots proposed by Müller et al. is the simplest one to estimate approximate values.[^46^, ^47^] Here, by setting the propagating radical concentration at 10^−7^ M as in a similar way to the papers by Moad et al,[^44^, ^45^] *C*_tr_ values were estimated for the polymerizations in PhC(CF_3_)_2_OH at 60 °C (Figure 2) as shown in Figure S9. The *C*_tr_ value of **P_2_** was 0.21, which was close to the reported value (0.19)^[26]^ irrespective of the different conditions. The values of a series of *exo*‐olefin‐based chain‐transfer agents were thus estimated as follows: 0.22 (**3**), 0.40 (**1**), 0.41 (**2**), 0.42 (**5**), 0.62 (**4**), 1.50 (**6**). These results indicate again that **4** and **6** are relatively efficient chain‐transfer agents.

**Figure 3:**
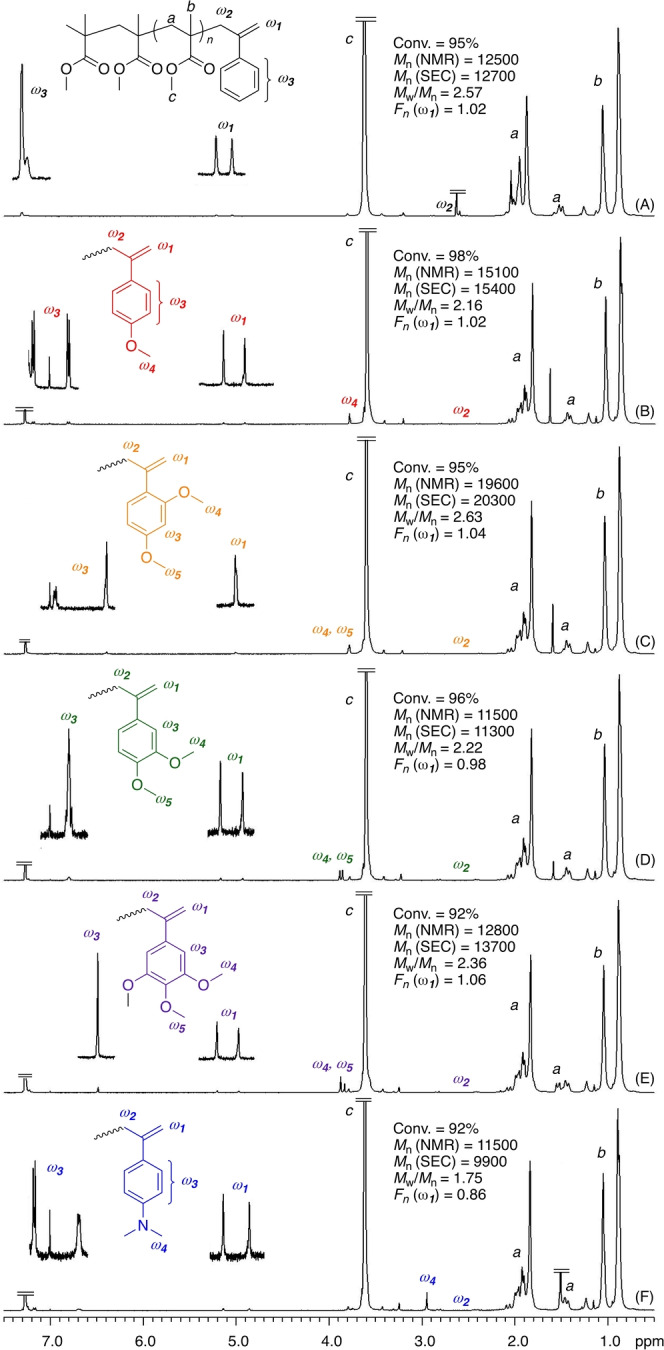
^1^H NMR spectra ((CD_3_)_2_CO, 50 °C for (A), CDCl_3_, 55 °C for (B)–(F)) of PMMA obtained i radical polymerizations of MMA in the presence of sulfur‐free RAFT agents (**1** (A), **2** (B), **3** (C), **4** (D), **5** (E) **6** (F)).

The ^1^H NMR spectra of all of the polymers obtained with **1**–**6** showed the characteristic peaks of the −CH_2_C(=CH_2_)Ph−Y groups, such as methylene, phenyl, methoxy, and dimethylamino, at the ω‐chain end (Figure 3). The *M*_n_ values (*M*_n_(NMR)) calculated from the peak intensity ratios of the chain‐end methylene protons (*e*) to the side‐chain methyl ester protons (*c*) were in good agreement with those measured by size‐exclusion chromatography (SEC) using PMMA as the standard (*M*_n_(SEC)). These results indicate that all of the obtained PMMA chains nearly quantitatively possess −CH_2_C(=CH_2_)Ph−Y termini, which were formed via the RAFT mechanism. In addition, the obtained polymers are new end‐functionalized PMMAs with various substituted styrene groups, which can be used as macro‐RAFT agents for the radical polymerization of methacrylates (see below) or macromonomers for radical copolymerization with other mono‐substituted vinyl monomers, such as acrylates and styrenes. The end functionality (*F_n_*) calculated from the ratio of *M*_n_(SEC) to *M*_n_(NMR) was thus close to unity. However, the *F_n_* obtained with **6**, with a dimethylamino group, was 0.86, suggesting the lower stability. In total, **4**, having a 3,4‐dimethoxystyrene terminus, is thus most effective as a sulfur‐free RAFT agent for the homogeneous radical polymerization of MMA.

Effects of polymerization temperature were also studied. The *M*_n_ values of the polymers obtained using 2,2′‐azobis(*N*‐butyl‐2‐methylpropionamide) (VAm‐110) in the presence of **2** at 100 °C in PhC(CF_3_)_2_OH were lower and closer to the calculated values than those at 60 °C (Figure S10). However, the chain‐end fidelity was decreased (*F_n_* (ω_1_)=0.91) in comparison to that obtained at 60 °C (*F_n_* (ω_1_)=0.98), suggesting that side reactions occurred at a higher temperature.

The PMMA obtained from **4** at 60 °C was then isolated by preparative SEC (*M*_n_=3200, *M*_w_/*M*_n_=1.92) and used as a macro‐RAFT agent for the second radical polymerization of MMA in PhC(CF_3_)_2_OH at 60 °C. The polymerization occurred smoothly, and the conversion of MMA reached 91 % in 24 h (Figure S11A), resulting in polymers with higher molecular weights (*M*_n_=5400, *M*_w_/*M*_n_=1.70) (Figure S12). In particular, the *M*_n_ values increased in direct proportion to monomer conversion and agreed well with the calculated values.

Furthermore, block copolymerization was investigated using another PMMA obtained from **4** (*M*_n_=2900, *M*_w_/*M*_n_=1.95) as the macro‐RAFT agent with benzyl methacrylate (BzMA) as the second monomer under similar conditions. The conversion of BzMA reached 96 % in 27 h (Figure S11B), resulting in polymers with similarly higher molecular weights (*M*_n_=6500, *M*_w_/*M*_n_=1.81) (Figure S12). A similar linear increase in the *M*_n_ values was confirmed and in good agreement with the calculated vales.

The ^1^H NMR spectrum of the obtained polymer showed signals from both the MMA and BzMA units and the terminal 3,4‐dimethoxystyrene moiety, such as phenyl, methoxy, and methylene groups (Figure S13). In particular, the chemical shifts of the terminal phenyl and methylene groups changed slightly from the shifts of these groups when attached to PMMA as the macro‐RAFT agent, indicating that the −CH_2_C(=CH_2_)Ph−Y group was efficiently transferred to the poly(BzMA) terminal via RAFT block polymerization. The end functionality of the *exo*‐olefin group attached to the BzMA unit was 0.99 and close to unity. In addition, the number‐average degrees of polymerization (DP_*n*_) of MMA and BzMA calculated from the peak intensity ratios of the terminal *exo*‐olefin groups to the side chain methyl and benzyl ester groups were 29 and 18, which were close to the theoretical values, 29 and 19, respectively. Thus, PMMA with the terminal 3,4‐dimethoxystyrene unit quantitatively operates as a macro‐RAFT agent to produce block copolymers of MMA and BzMA.

Multiblock copolymerizations of methacrylates were further investigated by sequential monomer addition using a syringe pump, by which the monomers were slowly supplied to the homogeneous solutions to maintain the monomer concentrations at a relatively low level. This method could slow propagation relative to the chain‐transfer reaction and narrow the molecular weight distributions.

Triblock copolymerization of MMA, ethyl methacrylate (EMA), and *n*‐butyl methacrylate (BMA) was first conducted using **2** and AIBN in PhC(CF_3_)_2_OH at 60 °C. As shown in Figure S14, MMA, as the first monomer, was added to a solution of **2** and AIBN in PhC(CF_3_)_2_OH with a syringe pump at a constant rate for the first 11 h. MMA monomer conversion reached 93 % 16 h after the reaction started. The obtained polymers had a relatively narrow MWD (*M*_w_/*M*_n_=1.50) and controlled *M*_n_ (*M*_n_=2500), agreeing well with the calculated value (Figure S15). Similarly, EMA was added at a constant rate for 11 h. The polymer obtained after a total of 36 h had a narrower MWD (*M*_w_/*M*_n_=1.45) and controlled *M*_n_ (*M*_n_=4200), and the monomer conversions were ≥99 % and 91 % for MMA and EMA, respectively. Finally, BMA was added for 12 h. The MWD became even narrower (*M*_w_/*M*_n_=1.41), and the *M*_n_ (*M*_n_=6900) agreed well with the calculated value (conversions of MMA, EMA, and BMA: ≥99 %, ≥99 %, and 92 %, respectively). The polymers obtained by continuous monomer addition thus had more controlled molecular weights and narrower MWDs.

**Figure 4:**
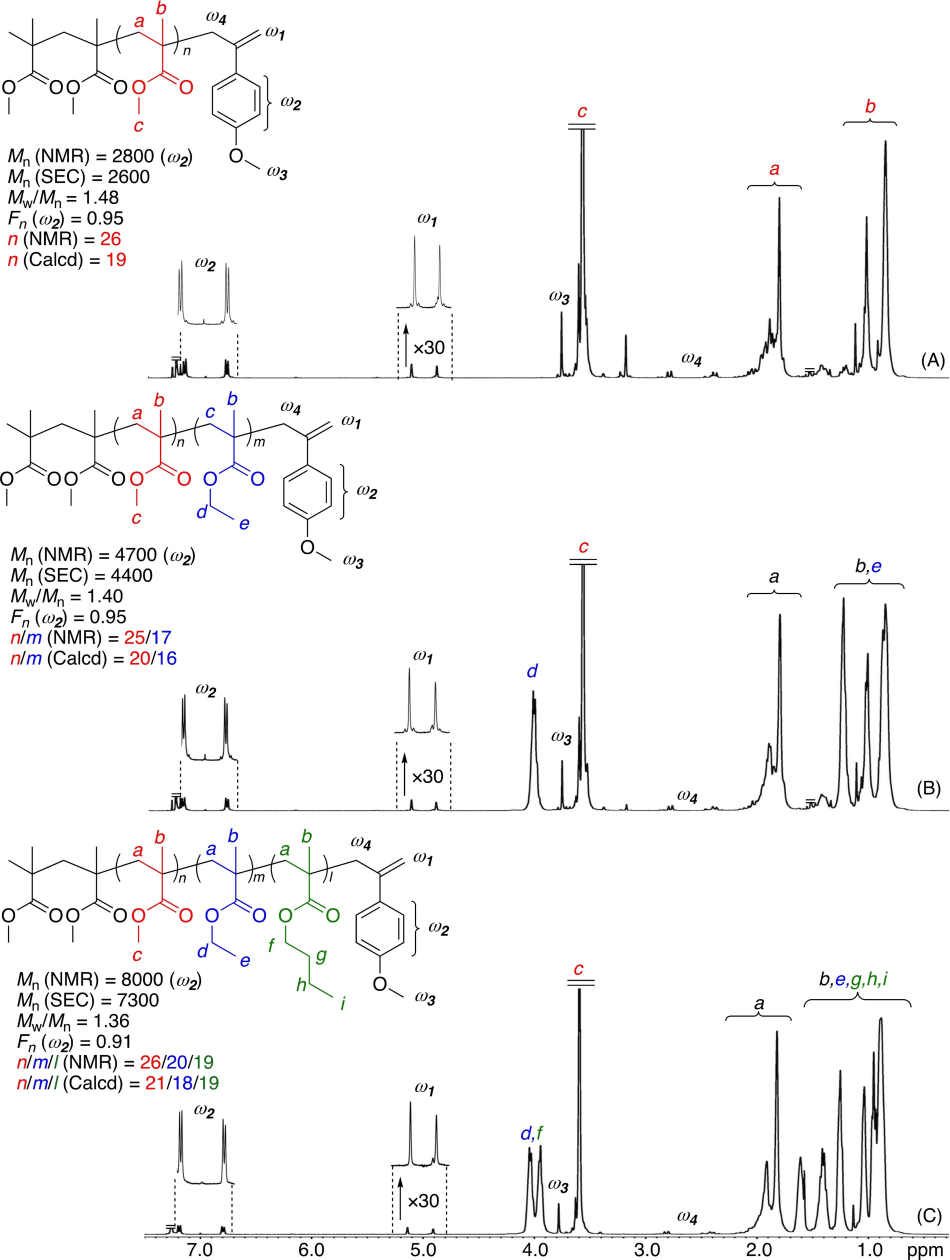
^1^H NMR spectra (CDCl_3_, 55 °C) of PMMA (A), PMMA‐*b*‐PEMA diblock copolymer (B), and PMMA‐*b*‐PEMA*‐b*‐PBMA triblock copolymer (C) obtained via continuous monomer additions using a syringe pump at 2.0 μL min^−1^ in PhC(CF_3_)_2_OH at 60 °C.: [MMA]_0_/[EMA]_add_/[BMA]_add_/[**2**]_0_/[AIBN]_0_=1000/1000/1000/50/20 mM.

The ^1^H NMR spectra of the polymers obtained after the addition of each monomer showed the characteristic alkyl protons of its pendant ester moiety (Figure 4). In addition, the protons from the *exo*‐olefin and phenyl groups at the ω‐chain ends were observed. The *M*_n_ values calculated from the peak intensity ratios of the *exo*‐olefin peaks to the side‐chain ester moieties were close to those measured by SEC. The end functionalities (*F_n_* =*M*_n_(SEC)/*M*_n_(NMR)) of the *exo*‐olefin groups were 0.95, 0.95, and 0.91 at each stage and thus close to unity. Furthermore, the DP_*n*_ values obtained from the ^1^H NMR spectra were close to the calculated values. These results indicate the formation of MMA, EMA, and BMA triblock copolymers using the sulfur‐free RAFT agent in homogeneous solutions.

**Figure 5:**
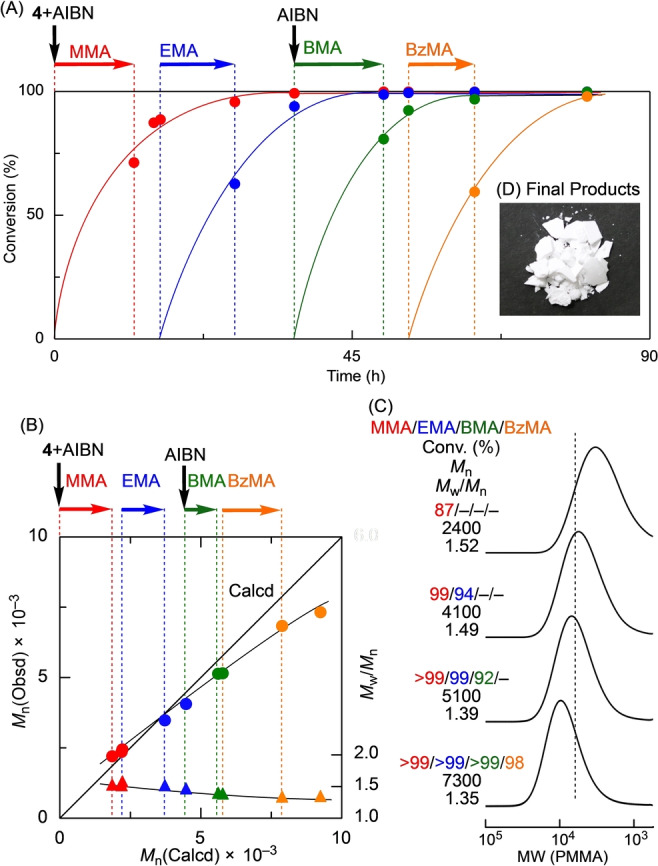
Time‐conversion curves (A), *M*_n_ values (B), SEC curves (C), and picture of polymers obtained via continuous monomer additions using a syringe pump at 2.0 μL min^−1^ in PhC(CF_3_)_2_OH at 60 °C: [MMA]_0_/[EMA]_add_/[BMA]_add_/[BzMA]_add_/[**4**]_0_=1000/1000/1000/1000/50 mM, [AIBN]_total_=[AIBN]_0_+[AIBN]_add_=20+20 mM.

Similar techniques were further used for the tetrablock polymerization of MMA, EMA, BMA, and BzMA with **4** and AIBN in PhC(CF_3_)_2_OH at 60 °C (Figure 5). The MWDs became narrower with monomer addition. The *M*_w_/*M*_n_ value finally decreased to its lowest value of 1.35, although the *M*_n_ values were slightly lower than the calculated values. The *F_n_* values obtained from the ^1^H NMR spectra (Figure S16) were 0.96, 0.96, 0.85, and 0.72, suggesting that some of the ω‐chain ends were gradually lost with the increasing number of monomer addition steps. However, more interestingly, the final products obtained by precipitation in methanol were completely colorless, as shown in the inset in Figure 5.

For reference, the tetrablock polymerizations of MMA, EMA, BMA, and BzMA were investigated by adding each of the polymers at once without using a syringe pump (Figure S17). In this case, the initial *M*_n_ values were apparently higher than the calculated values, but they became close to those values with monomer addition. In addition, the MWDs became narrower and finally reached *M*_w_/*M*_n_=1.52, which was slightly broader than that obtained with continuous monomer addition. A series of ^1^H NMR spectra also indicated the formation of tetrablock copolymers, although the *F_n_* values gradually decreased (1.01, 0.94, 0.84, and 0.83) (Figure S18).

## Conclusion

A series of new *exo*‐olefin compounds composed of styrenic groups and MMA dimer units worked as sulfur‐free RAFT agents for the homogeneous radical polymerization of methacrylates. Their performance as chain‐transfer agents was enhanced with incorporation of electron‐donating substituents on the phenyl group and was superior to that of the *exo*‐olefin MMA trimer, which was previously one of the most efficient sulfur‐free RAFT agents. The obtained polymers further act as macro‐RAFT agents to prepare methacrylate block copolymers. In addition, multiblock copolymers were obtained by sequential monomer addition, whereas continuous addition using a syringe pump was efficient for producing narrow MWDs. These sulfur‐free *exo*‐olefin‐based RAFT agents could contribute to further developments in controlled polymerization via the RAFT mechanism and in the design of polymer materials.

## Conflict of interest

The authors declare no conflict of interest.

1

## Supporting information

As a service to our authors and readers, this journal provides supporting information supplied by the authors. Such materials are peer reviewed and may be re‐organized for online delivery, but are not copy‐edited or typeset. Technical support issues arising from supporting information (other than missing files) should be addressed to the authors.

Supporting Information

## Data Availability

Research data are not shared.
