# Supplementary material for: Sulfur‐Free Radical RAFT Polymerization of Methacrylates in Homogeneous Solution: Design of exo‐Olefin Chain‐Transfer Agents (R−CH2C(=CH2)Z)
Source: Angew Chem Int Ed Engl. 2022 Nov 23;61(52):e202212633. doi: 10.1002/anie.202212633 (PMC10099145; doi:10.1002/anie.202212633)
Supplement: Supplementary file 1 — Supporting Information [file ANIE-61-0-s001.pdf]

Angewandte Chemie
Eine Zeitschrift der Gesellschaft Deutscher Chemiker

Supporting Information

Sulfur-Free Radical RAFT Polymerization of Methacrylates in Homogeneous Solution: Design of *exo*-Olefin Chain-Transfer Agents ($R{-}CH_2C({=}CH_2)Z$)

M. Amano, M. Uchiyama, K. Satoh, M. Kamigaito**

Supporting Information

Contents:

Experimental Section ········· S2
Figure S1 ········· S12
Figure S2 ········· S13
Figure S3 ········· S14
Figure S4 ········· S14
Figure S5 ········· S15
Figure S6 ········· S15
Figure S7 ········· S16
Figure S8 ········· S16
Figure S9 ········· S17
Figure S10 ········· S18
Figure S11 ········· S18
Figure S12 ········· S19
Figure S13 ········· S19
Figure S14 ········· S20
Figure S15 ········· S20
Figure S16 ········· S21
Figure S17 ········· S22
Figure S18 ········· S23

Supporting Information

Experimental Section

Materials

Methyl methacrylate (MMA) (TCI, >99.8%), ethyl methacrylate (EMA) (TCI, >99.0%), *n*-butyl methacrylate (BMA) (TCI, >99.0%), benzyl methacrylate (BzMA) (TCI, >98.0%), $PhC(CF_3)_2OH$ (FUJIFILM Wako, 98.0%), dimethyl sulfoxide (KANTO, >99.0%), 1,2,3,4-tetrahydronaphthalene (tetralin) (FUJIFILM Wako, 97%), and 2-butanone (TCI, >99.0%) were distilled over calcium hydride under reduced pressure before use. Acetophenone (TCI, 98.5%), 4'-methoxyacetophenone (TCI, >99.0%), 2',4'-dimethoxyacetophenone (TCI, >96.0%), 3',4'-dimethoxyacetophenone (TCI, >98.0%), 3',4',5'-trimethoxyacetophenone (TCI, >98.0%), 4'-dimethylaminoacetophenone (TCI, >98.0%), chlorotrimethylsilane (TCI, >98.0%), acetonitrile (KANTO, >99.5%), trimethyl amine (TCI, >99.0%), CuCl (Sigma-Aldrich, 99.99%), 2,2'-bipyridine (bpy) (Sigma-Aldrich, >99%), (methyl)triphenylphosphonium bromide (KANTO, >98%), *n*-butyl lithium (KANTO, 2.65 M solution in *n*-hexane), Tebbe reagent (Sigma-Aldrich, 0.5 M solution in toluene), and ammonium chloride (Kishida, >99.5%) were used as received. 2,2'-Azobisisobutyronitrile (AIBN) (Kishida, >99%) and 2,2'-azobis(*N*-butyl-2-methylpropionamide) (VAm-110) (FUJIFILM Wako, >95%) were purified by recrystallization from methanol. Dimethyl-2,2'-azobis(isobutyrate) (MAIB) (FUJIFILM Wako, 97.0%) was purified by recrystallization from acetone. MMA dimer olefin (**P**$_1$: $CH_3C(CH_3)(CO_2Me)–CH_2C(=CH_2)CO_2Me$),[1] MMA dimer chloride (H–(MMA)$_2$–Cl: $H–(CH_2C(CH_3)(CO_2Me))_2–Cl$),[2] and difluoroborylbis(dimethylglyoximato)cobalt(II) dihydrate ($Co(dmgBF_2)_2(H_2O)_2$)[3] were prepared according to the literature. Toluene (KANTO, >99.5%; H_2O <10 ppm) and tetrahydrofuran (THF) (KANTO, >99.5%; H_2O <0.001% ppm) were dried and deoxygenated by passage through columns of a Glass Contour System before use.

Synthesis of Dimethyl-2,2,4-trimethyl-4-(2-phenylallyl)pentanedioate (1)

Dimethyl-2,2,4-trimethyl-4-(2-phenylallyl)pentanedioate (**1**) was synthesized by the following procedures. α-(Trimethylsiloxy)styrene was first synthesized by reaction between acetophenone and chlorotrimethylsilane. Chlorotrimethylsilane (150 mL, 1.19 mol) was added dropwise to a mixture of acetophenone (194 mL, 977 mmol), sodium iodide (169 g, 1.13 mol), triethylamine (167 mL, 1.2 mol), and acetonitrile (822 mL) in a 2 L three-necked round-bottom flask at room temperature. After stirring for 1 h, the solvent was removed under vacuum. Then, dry *n*-hexane (500 mL) was added to the residue to precipitate the salts, and the salts were filtered off. This process was repeated three times. The filtrate was evaporated under reduced pressure to remove the *n*-hexane. After purification by distillation under reduced pressure (1300 Pa, bp. 90 °C), α-(trimethylsiloxy)styrene was obtained as a colorless liquid (169 g, 878 mmol,

yield = 90%).

Then, dimethyl 2,2,4-trimethyl-4-(2-oxo-2-phenylethyl)pentanedioate (**1’**) was synthesized by the radical addition reaction between H–(MMA)$_2$–Cl and α-(trimethylsiloxy)styrene. CuCl (0.148 g, 1.49 mmol), 2,2’-bipyridine (0.467 g, 2.99 mmol), and dimethyl sulfoxide (26 mL) were placed in a 100 mL round-bottom flask under dry argon. The catalyst solution was stirred for 1 h at 20 °C. Then, the reaction was initiated by the addition of a mixture of H–(MMA)$_2$–Cl (1.41 g, 5.97 mmol), α-(trimethylsiloxy)styrene (5.75 mL, 29.9 mmol), and dimethyl sulfoxide (26 mL) to the catalyst solution at 20 °C. After 24 h, the reaction mixture was quenched by cooling to –78 °C. The product was extracted with toluene and washed with diluted hydrochloric acid and distilled water. The solvent was removed by evaporation to give the crude product. After purification by column chromatography on silica gel with *n*-hexane/ethyl acetate (9/1) as the eluent, **1’** was obtained (1.92 g, 5.99 mmol, yield >99%, purity >98%).

Finally, **1** was synthesized by the Wittig reaction of **1’**. *n*-BuLi (3.70 mL of a 2.65 M *n*-hexane solution, 9.8 mmol) was added dropwise to a suspension of (methyl)triphenylphosphonium bromide (3.64 g, 10.2 mmol) and THF (20 mL) in a 100 mL round-bottom flask at –78 °C. The mixture was warmed to 0 °C and stirred for 1 h. The mixture was then cooled again to –78 °C, and then a solution of **1’** (1.58 g, 4.93 mmol) in THF (22 mL) was added dropwise. The reaction mixture was gradually warmed to 20 °C. After 20 h, the reaction was quenched by the addition of saturated aqueous NH_4Cl solution. The product was extracted with Et_2O and washed with distilled water. The solvent was removed by evaporation to give the crude product. After purification by column chromatography on silica gel with *n*-hexane/ethyl acetate (9/1) as the eluent, **1** was obtained as a white solid (645 mg, 2.03 mmol, yield = 41%).

Synthesis of Dimethyl-2-(2-(4-methoxyphenyl)allyl)-2,4,4-trimethylpentanedioate (2)

Dimethyl-2-(2-(4-methoxyphenyl)allyl)-2,4,4-trimethylpentanedioate (**2**) was synthesized by the following procedures. 4-Methoxy-α-(trimethylsiloxy)styrene was first synthesized by the reaction between 4’-methoxyacetophenone and chlorotrimethylsilane. Chlorotrimethylsilane (17.2 mL, 158 mmol) was added dropwise to a mixture of 4’-methoxyacetophenone (16.8 g, 112 mmol), sodium iodide (19.4 g, 129 mmol), triethylamine (19.0 mL, 136 mmol), and acetonitrile (100 mL) in a 500 mL three-necked round-bottom flask at room temperature. After stirring for 2 h, the solvent was removed under vacuum. Then, dry *n*-hexane (100 mL) was added to the residue to precipitate the salts, and the salts were filtered off. This process was repeated three times. The filtrate was evaporated under reduced pressure to remove the *n*-hexane. After purification by distillation under reduced pressure (52 Pa, bp. 62 °C), 4-methoxy-α-(trimethylsiloxy)styrene was obtained as a colorless liquid (23.3 g, 105

mmol, yield = 94%).

Then, dimethyl 2-(2-(4-methoxyphenyl)-2-oxoethyl)-2,4,4-trimethylpentanedioate (**2’**) was synthesized by the radical addition reaction between H–(MMA)$_2$–Cl and 4-methoxy-α-(trimethylsiloxy)styrene. CuCl (245 mg, 2.46 mmol), 2,2’-bipyridine (773 mg, 4.95 mmol), and dimethyl sulfoxide (42 mL) were placed in a 200 mL round-bottom flask under dry argon. The catalyst solution was stirred for 1 h at 20 °C. Then, the reaction was initiated by the addition of a mixture of H–(MMA)$_2$–Cl (2.34 g, 9.89 mmol), 4-methoxy-α-(trimethylsiloxy)styrene (11.0 mL, 49.5 mmol), and dimethyl sulfoxide (42 mL) to the catalyst solution at 20 °C. After 24 h, the reaction mixture was quenched by cooling to –78 °C. The product was extracted with toluene and washed with diluted hydrochloric acid and distilled water. The solvent was removed by evaporation to give the crude product. After purification by column chromatography on silica gel with *n*-hexane/ethyl acetate (8/2) as the eluent, **2’** was obtained (3.15 g, 8.99 mmol, yield = 91%).

Finally, **2** was synthesized by the Wittig reaction of **2’**. *n*-BuLi (3.02 mL of a 2.65 M *n*-hexane solution, 8.0 mmol) was added dropwise to a suspension of (methyl)triphenylphosphonium bromide (3.00 g, 6.88 mmol) and THF (30 mL) in a 100 mL round-bottom flask at –78 °C. The mixture was warmed to 0 °C and stirred for 1 h. The mixture was cooled to –78 °C again, and then **2’** (3.86 mL of 104 mM THF solution, 4.01 mmol) was added dropwise. The reaction mixture was gradually warmed to 20 °C. After 22 h, the reaction was quenched by the addition of saturated aqueous NH_4Cl solution. The product was extracted with Et_2O and washed with distilled water. The solvent was removed by evaporation to give the crude product. After purification by column chromatography on silica gel with *n*-hexane/ethyl acetate (8/2) as the eluent, **2** was obtained as a white solid (462 mg, 1.33 mmol, yield = 33%).

Synthesis of Dimethyl-2-(2-(2,4-dimethoxyphenyl)allyl)-2,4,4-trimethylpentanedioate (3).

Dimethyl-2-(2-(2,4-dimethoxyphenyl)allyl)-2,4,4-trimethylpentanedioate (**3**) was synthesized by the following procedures. 2,4-Dimethoxy-α-(trimethylsiloxy)styrene was first synthesized by the reaction between 2’,4’-dimethoxyacetophenone and chlorotrimethylsilane. Chlorotrimethylsilane (21.3 mL, 169 mmol) was added dropwise to a mixture of 2’,4’-dimethoxyacetophenone (25.0 g, 139 mmol), sodium iodide (24.1 g, 161 mmol), triethylamine (23.6 mL, 169 mmol), and acetonitrile (125 mL) in a 300 mL three-necked round-bottom flask at room temperature. After stirring for 17 h, the solvent was removed under vacuum. Then, dry *n*-hexane (125 mL) was added to the residue to precipitate the salts, and the salts were filtered off. This process was repeated three times. The filtrate was evaporated under reduced pressure to remove the *n*-hexane. After purification by distillation under reduced pressure (21 Pa, bp. 60 °C), 2,4-dimethoxy-α-(trimethylsiloxy)styrene was obtained as a colorless liquid (20.9 g, 82.8 mmol, yield = 60%).

Then, dimethyl 2-(2-(2,4-dimethoxyphenyl)-2-oxoethyl)-2,4,4-trimethylpentanedioate (**3'**) was synthesized by the radical addition reaction between H–(MMA)$_2$–Cl and 2,4-dimethoxy-α-(trimethylsiloxy)styrene. CuCl (353 mg, 3.57 mmol), 2,2'-bipyridine (1.11 g, 7.13 mmol), and dimethyl sulfoxide (60 mL) were placed in a 200 mL round-bottom flask under dry argon. The catalyst solution was stirred for 1 h at 20 °C. Then, the reaction was initiated by the addition of a mixture of H–(MMA)$_2$–Cl (3.38 g, 14.3 mmol), 2,4-dimethoxy-α-(trimethylsiloxy)styrene (18.0 mL, 71.3 mmol), and dimethyl sulfoxide (60 mL) to the catalyst solution at 20 °C. After 22 h, the reaction mixture was quenched by cooling to –78 °C. The product was extracted with toluene and washed with diluted hydrochloric acid and distilled water. The solvent was removed by evaporation to give the crude product. After purification by column chromatography on silica gel with *n*-hexane/ethyl acetate (8/2) as the eluent, **3'** was obtained (3.78 g, 9.94 mmol, yield = 69%).

Finally, **3** was synthesized by the methylenation reaction of **3'**. Tebbe reagent (17.2 mL of 500 mM toluene solution, 8.60 mmol) was added dropwise to a mixture of **3'** (3.27 g, 8.60 mmol) and toluene (17 mL) in a 100 mL round-bottom flask at –78 °C. The reaction mixture was gradually warmed to room temperature. After 48 h, the reaction was quenched by slowly adding saturated aqueous $NaHCO_3$ solution. The product was extracted with Et_2O and washed with saturated aqueous $NaHCO_3$ solution and distilled water. The solvent was removed by evaporation to give the crude product. After purification by column chromatography on silica gel with *n*-hexane/ethyl acetate (7/1) as the eluent, **3** was obtained as a white solid (1.14 g, 3.01 mmol, yield = 35%).

Synthesis of Dimethyl-2-(2-(3,4-dimethoxyphenyl)allyl)-2,4,4-trimethylpentanedioate (4).

Dimethyl-2-(2-(3,4-dimethoxyphenyl)allyl)-2,4,4-trimethylpentanedioate (**4**) was synthesized by the following procedures. 3,4-Dimethoxy-α-(trimethylsiloxy)styrene was first synthesized by the reaction between 3',4'-dimethoxyacetophenone and chlorotrimethylsilane. Chlorotrimethylsilane (21.3 mL, 169 mmol) was added dropwise to a mixture of 3',4'-dimethoxyacetophenone (25.0 g, 139 mmol), sodium iodide (24.1 g, 161 mmol), triethylamine (23.6 mL, 169 mmol), and acetonitrile (125 mL) in a 300 mL three-necked round-bottom flask at room temperature. After stirring for 20 h, the solvent was removed under vacuum. Then, dry *n*-hexane (125 mL) was added to the residue to precipitate the salts, and the salts were filtered off. This process was repeated three times. The filtrate was evaporated under reduced pressure to remove the *n*-hexane. After purification by distillation under reduced pressure (47 Pa, bp. 113 °C), 3,4-dimethoxy-α-(trimethylsiloxy)styrene was obtained as a colorless liquid (20.1 g, 79.6 mmol, yield = 57%).

Then, dimethyl 2-(2-(3,4-dimethoxyphenyl)-2-oxoethyl)-2,4,4-trimethylpentanedioate (**4'**) was synthesized by the radical addition reaction between H–(MMA)$_2$–Cl and 3,4-dimethoxy-

α-(trimethylsiloxy)styrene. CuCl (373 mg, 3.77 mmol), 2,2'-bipyridine (1.18 g, 7.53 mmol), and dimethyl sulfoxide (63 mL) were placed in a 200 mL round-bottom flask under dry argon. The catalyst solution was stirred for 1 h at 20 °C. Then, the reaction was initiated by the addition of a mixture of H–(MMA)$_2$–Cl (3.56 g, 15.1 mmol), 3,4-dimethoxy-α-(trimethylsiloxy)styrene (19.0 mL, 75.3 mmol), and dimethyl sulfoxide (65 mL) to the catalyst solution at 20 °C. After 25 h, the reaction mixture was quenched by cooling to –78 °C. The product was extracted with toluene and washed with diluted hydrochloric acid and distilled water. The solvent was removed by evaporation to give the crude product. After purification by column chromatography on silica gel with *n*-hexane/ethyl acetate (8/2) as the eluent, **4'** was obtained (5.54 g, 14.6 mmol, yield = 96%).

Finally, **4** was synthesized by the Wittig reaction of **4'**. *n*-BuLi (11.0 mL of a 2.65 M *n*-hexane solution, 29.2 mmol) was added dropwise to a suspension of (methyl)triphenylphosphonium bromide (10.9 g, 30.6 mmol) and THF (59 mL) in a 200 mL round-bottom flask at –78 °C. The mixture was warmed to 0 °C and stirred for 1 h. The mixture was again cooled to –78 °C, and then a solution of **4'** (5.54 g, 14.6 mmol) in THF (59 mL) was added dropwise. The reaction mixture was gradually warmed to 20 °C. After 45 h, the reaction was quenched by the addition of saturated aqueous NH_4Cl solution. The product was extracted with Et_2O and washed with distilled water. The solvent was removed by evaporation to give the crude product. After purification by column chromatography on silica gel with *n*-hexane/ethyl acetate (7/3) as the eluent, **4** was obtained as a white solid (1.17 g, 3.09 mmol, yield = 21%).

Synthesis of Dimethyl-2-(2-(3,4,5-trimethoxyphenyl)allyl)-2,4,4-trimethylpentanedioate (5)

Dimethyl-2-(2-(3,4,5-trimethoxyphenyl)allyl)-2,4,4-trimethylpentanedioate (**5**) was synthesized by the following procedures. 3,4,5-Trimethoxy-α-(trimethylsiloxy)styrene was first synthesized by the reaction between 3',4',5'-trimethoxyacetophenone and chlorotrimethylsilane. Chlorotrimethylsilane (21.2 mL, 168 mmol) was added dropwise to a mixture of 3',4',5'-trimethoxyacetophenone (25.0 g, 119 mmol), sodium iodide (20.5 g, 137 mmol), triethylamine (20.1 mL, 144 mmol), and acetonitrile (100 mL) in a 200 mL three-necked round-bottom flask at room temperature. After stirring for 5.5 h, the solvent was removed under vacuum. Then, dry *n*-hexane (100 mL) was added to the residue to precipitate the salts, and the salts were filtered off. This process was repeated three times. The filtrate was evaporated under reduced pressure to remove the *n*-hexane. After purification by distillation under reduced pressure (23 Pa, bp. 93 °C), 3,4,5-trimethoxy-α-(trimethylsiloxy)styrene was obtained as a colorless liquid (27.7 g, 98.0 mmol, yield = 82%).

Then, dimethyl 2,2,4-trimethyl-4-(2-oxo-2-(3,4,5-trimethoxyphenyl)ethyl)pentanedioate (**5'**) was synthesized by the radical addition reaction between H–(MMA)$_2$–Cl and 3,4,5-

trimethoxy-α-(trimethylsiloxy)styrene. CuCl (535 mg, 5.40 mmol), 2,2'-bipyridine (1.70 g, 10.9 mmol), and dimethyl sulfoxide (94 mL) were placed in a 300 mL round-bottom flask under dry argon. The catalyst solution was stirred for 1 h at 20 °C. Then, the reaction was initiated by the addition of a mixture of H–(MMA)$_2$–Cl (5.14 g, 21.7 mmol), 2,4-dimethoxy-α-(trimethylsiloxy)styrene (24.5 mL, 86.8 mmol), and dimethyl sulfoxide (94 mL) to the catalyst solution at 20 °C. After 24 h, the reaction mixture was quenched by cooling to –78 °C. The product was extracted with toluene and washed with dilute hydrochloric acid and distilled water. The solvent was removed by evaporation to give the crude product. After purification by column chromatography on silica gel with ethyl acetate as the eluent, **5'** was obtained (6.60 g, 16.1 mmol, yield = 74%).

Finally, **5** was synthesized by the methylenation reaction of **5'**. Tebbe reagent (17.8 ml of a 500 mM toluene solution, 8.90 mmol) was added dropwise to a mixture of **5'** (3.60 g, 8.92 mmol) and toluene (18 mL) in a 100 mL round-bottom flask at –78 °C. The reaction mixture was gradually warmed to room temperature. After 47 h, the reaction was quenched by slowly adding saturated aqueous $NaHCO_3$ solution. The product was extracted with Et_2O and washed with saturated aqueous $NaHCO_3$ solution and distilled water. The solvent was removed by evaporation to give the crude product. After purification by column chromatography on silica gel with *n*-hexane/ethyl acetate (3/1) as the eluent, **5** was obtained as a white solid (470 mg, 1.15 mmol, yield = 13%).

Synthesis of Dimethyl-2-(2-(4-(dimethylamino)phenyl)allyl)-2,4,4-trimethylpentanedioate (6).

Dimethyl-2-(2-(4-(dimethylamino)phenyl)allyl)-2,4,4-trimethylpentanedioate (**6**) was synthesized by the following procedures. 4-Dimethylamino-α-(trimethylsiloxy)styrene was first synthesized by the reaction between 4'-dimethylaminoacetophenone and chlorotrimethylsilane. Chlorotrimethylsilane (23.5 mL, 186 mmol) was added dropwise to a mixture of 4'-dimethylaminoacetophenone (25.0 g, 153 mmol), sodium iodide (26.6 g, 177 mmol), triethylamine (25.9 mL, 186 mmol), and acetonitrile (140 mL) in a 500 mL three-necked round-bottom flask at room temperature. After stirring for 16 h, the solvent was removed under vacuum. Then, dry *n*-hexane (125 mL) was added to the residue to precipitate the salts, and the salts were filtered off. This process was repeated three times. The filtrate was evaporated under reduced pressure to remove the *n*-hexane. After purification by distillation under reduced pressure (24 Pa, bp. 79 °C), 4-dimethylamino-α-(trimethylsiloxy)styrene was obtained as a pale yellow liquid (15.3 g, 65.0 mmol, yield = 42%).

Then, dimethyl 2-(2-(4-(dimethylamino)phenyl)allyl)-2,4,4-trimethylpentanedioate (**6'**) was synthesized by the radical addition reaction between H–(MMA)$_2$–Cl and 4-dimethylamino-α-(trimethylsiloxy)styrene. CuCl (284 mg, 2.87 mmol), 2,2'-bipyridine (895 mg, 5.73 mmol), and

dimethyl sulfoxide (49 mL) were placed in a 200 mL round-bottom flask under dry argon. The catalyst solution was stirred for 1 h at 20 °C. Then, the reaction was initiated by the addition of a mixture of H–$(MMA)_2$–Cl (2.71 g, 11.4 mmol), 4-dimethylamino-α-(trimethylsiloxy)styrene (13.5 mL, 57.3 mmol), and dimethyl sulfoxide (49 mL) to the catalyst solution at 20 °C. After 24 h, the reaction mixture was quenched by cooling to –78 °C. The product was extracted with toluene and washed with dilute hydrochloric acid and distilled water. The solvent was removed by evaporation to give the crude product. After purification by column chromatography on silica gel with *n*-hexane/ethyl acetate (8/2) as the eluent, **6'** was obtained (3.55 g, 9.74 mmol, yield = 86%).

Finally, **6** was synthesized by the Wittig reaction of **6'**. *n*-BuLi (6.98 mL of a 2.65 M *n*-hexane solution, 8.00 mmol) was added dropwise to a suspension of (methyl)triphenylphosphonium bromide (6.93 g, 19.4 mmol) and THF (38 mL) in a 100 mL round-bottom flask at –78 °C. The mixture was warmed to 0 °C and stirred for 1 h. The mixture was cooled again to –78 °C, and then a solution of **6'** (3.37 g, 9.25 mmol) in THF (38 mL) was added dropwise. The reaction mixture was gradually warmed to 20 °C. After 23 h, the reaction was quenched by the addition of saturated aqueous NH_4Cl solution. The product was extracted with Et_2O and washed with distilled water. The solvent was removed by evaporation to give the crude product. After purification by column chromatography on silica gel with *n*-hexane/ethyl acetate (8/2) as the eluent, **6** was obtained as a pale yellow liquid (1.06 g, 2.93 mmol, yield = 32%).

Synthesis of 2,4,6-Tris(methoxycarbonyl)-4,6-dimethylhept-1-ene (P_2)

2,4,6-Tris(methoxycarbonyl)-4,6-dimethylhept-1-ene (**P_2**) was synthesized by the following procedures. MAIB (2.71 g, 11.8 mmol) and $Co(dmgBF_2)_2(H_2O)_2$ (0.723 g 1.72 mmol) were placed in a 1 L three-necked round-bottom flask with a three-way stopcock. After degassing, 2-butanone (250 mL) and MMA (250 mL, 2.34 mol) were added via dry syringes. The reaction solution was mixed and then heated under reflux (80 °C). After 33 h, the reaction was quenched by cooling the reaction mixture to –78 °C. The solvent and residual MMA were removed by evaporation to give the crude product, which was a mixture of dimers, trimers, and other oligomers. The dimers were removed by distillation under reduced pressure (19 Pa, bp. 48 °C). Then, the residue was purified by column chromatography on silica gel with $CHCl_3$ as the eluent to remove the cobalt catalyst and other oligomers. The solvent was removed by evaporation to give the product mainly containing the trimer (**P_2**). After purification by distillation (19 Pa, bp. 89 °C), **P_2** was obtained as a colorless liquid (14.7 g, 49.1 mmol, yield = 4%).

Radical RAFT Polymerization of MMA

Radical RAFT polymerization was carried out by the syringe technique under dry nitrogen in sealed glass tubes. A typical example of the reaction procedure is given below. MMA (1.75 mL, 17.5 mmol), **2** (0.84 mL of a 208 mM solution in toluene, 0.17 mmol), AIBN (0.18 mL of a 100 mM $PhC(CF_3)_2OH$ solution, 0.018 mmol), tetlrain (0.05 mL) and $PhC(CF_3)_2OH$ (0.68 mL) were placed in a 25 mL round-bottom flask equipped with a three-way stopcock. The total volume of the reaction mixture was 3.5 mL. Immediately after mixing, the solution was evenly charged in seven glass tubes, which were then flame-sealed under a nitrogen atmosphere. The tubes were immersed in a thermostatic oil bath at 60 °C. At predetermined intervals, the polymerization was terminated by cooling the reaction mixture to –78 °C. Monomer conversion was determined from the concentration of monomer measured by ^{1}H NMR with tetralin as an internal standard (35 h, 98%). The quenched reaction solution was evaporated to dryness to give the product PMMA (M_n = 14300, M_w/M_n = 2.35).

Block Polymerization via Continuous Addition Using a Syringe Pump

A typical example of block copolymerization with continuous monomer addition using a syringe pump under an argon atmosphere is given below. First, **2** (215 mg, 0.619 mmol), AIBN (1.24 mL of a 200 mM solution in $PhC(CF_3)_2OH$, 0.248 mmol), and $PhC(CF_3)_2OH$ (9.62 mL) were placed in a 50 mL two-neck round-bottom flask equipped with a three-way stopcock. After mixing, the flask was immersed in a thermostatic oil bath at 60 °C. Then, MMA (1.33 mL, 12.4 mmol) was added to the reaction mixture using a syringe pump at 2.0 μL min^{-1} over 11 h. The weight of the reaction solution was 17.56 g when the addition was complete. At predetermined intervals, an aliquot of the reaction mixture was sampled to monitor monomer conversion. Sixteen hours after MMA addition began, MMA conversion had reached 93%. The weight of the reaction mixture decreased from 17.56 g to 14.98 g (85% reduction) by sampling after MMA addition had completed. After 0.5 h, EMA (1.31 mL, 10.5 mmol) was added to the reaction mixture using a syringe pump at 2.0 μL min^{-1} over 11 h. The weight of the reaction solution was 16.11 g when the addition was complete. EMA conversion reached 91% at 19.5 h after addition began. The weight of the reaction mixture was reduced from 16.11 g to 13.78 g (85% reduction) by sampling after EMA addition had completed. After 0.5 h, BMA (1.43 mL, 9.00 mmol) was added to the reaction mixture using a syringe pump at 2.0 μL min^{-1} over 12 h. The weight of the reaction solution was 15.02 g when the addition was complete. Twenty-eight hours after the start of BMA addition, BMA conversion had reached 92%. Then, the reaction was quenched by cooling to –78 °C. Monomer conversion was determined from the integral ratios of vinylidene groups to side groups of both monomers and polymers measured by ^{1}H NMR (64.5 h, MMA >99%, EMA >99%, BMA = 92%). The quenched reaction solution was evaporated to dryness to give the multiblock copolymer (M_n = 6900, M_w/M_n = 1.41).

Calculation of Chain-Transfer Constant (C_{tr}).

Approximate chain-transfer constants (C_{tr}) of chain-transfer agents (CTA) were estimated from M_n or P_n (P_n: number-average degree of polymerization) vs conversion plots (Figure S9) according to the following equations:[4,5]

$$P_n = \frac{bx}{1-(1-a)(1-x)^{C_{tr}}}$$

$$M_n = \frac{bx}{1-(1-a)(1-x)^{C_{tr}}}\mathrm{MW(monomer)} + \mathrm{MW(CTA)}$$

where x is the monomer conversion, a is [active species]$_0$/[CTA]$_0$, and b is [monomer]$_0$/[CTA]$_0$. Here, the propagating radical concentration was set at 10^{-7} M as in a similar way to the reported papers.[6,7]

Measurements

Monomer conversion was determined from the concentration of residual monomer measured by ^{1}H NMR spectroscopy with tetralin as an internal standard. ^{1}H and ^{13}C NMR spectra were recorded on a JEOL ECS-400 spectrometer operating at 400 MHz. The number-average molecular weight (M_n) and the molecular weight distribution (M_w/M_n) of the product polymers were determined by size-exclusion chromatography (SEC) in THF at 40 °C on two polystyrene gel columns [Shodex KF-805 L (pore size: 20–1000 Å; 8.0 mm i.d. × 30 cm) × 2] connected to a JASCO PU-2080 precision pump and JASCO RI-2031 detector. The columns were calibrated against 10 standard poly(methyl methacrylate) samples (Agilent Technology; M_p = 202–1677000, M_w/M_n = 1.02–1.09).

References

1. Haddleton, D. M.; Maloney, D. R.; Suddaby, K. G. Competition between β-Scission of Macromonomer-Ended Radicals and Chain Transfer to Cobalt(II) in Catalytic Chain Transfer Polymerization (CCTP). *Macromolecules* **1996**, *29*, 481–483.
2. Ando, T.; Kamigaito, M.; Sawamoto, M. Reversible Activation of Carbon−Halogen Bonds by $RuCl_2(PPh_3)_3$: Halogen Exchange Reactions in Living Radical Polymerization. *Macromolecules* **2000**, *33*, 2819–2824.
3. Bakac, A.; Brynildson, M. E.; Espenson, J. H. Characterization of the Structure, Properties, and Reactivity of a Cobalt(II) Macrocyclic Complex. *Inorg. Chem.* **1986**, *25*, 4108–4114.
4. Müller, A. H. E.; Zhuang, R.; Yan, D.; Litvinenko, G. Kinetic Analysis of "Living" Polymerization Processes Exhibiting Slow Equilibria. 1. Degenerative Transfer (Direct Activity Exchange between Active and "Dormant" Species). Application to Group Transfer Polymerization. *Macromolecules* **1995**, *28*, 4326–4333.

5. Litvinenko, G.; Müller, A. H. E. General Kinetic Analysis and Comparison of Molecular Weight Distributions for Various Mechanisms of Activity Exchange in Living Polymerizations. *Macromolecules* **1997**, *30*, 1253–1266.
6. Chong, Y. K.; Krstina, J.; Le, T. P. T.; Moad, G.; Postma, A.; Rizzardo, E.; Thang, S. H. Thiocarbonylthio Compounds [SC(Ph)S−R] in Free Radical Polymerization with Reversible Addition-Fragmentation Chain Transfer (RAFT Polymerization). Role of the Free-Radical Leaving Group (R). *Macromolecules* **2003**, *36*, 2256–2272.
7. Moad, G.; Rizzardo, E.; Thang, S. H. Radical Addition–Fragmentation Chemistry in PolymerSynthesis. *Polymer* **2008**, *49*, 1079–1131.

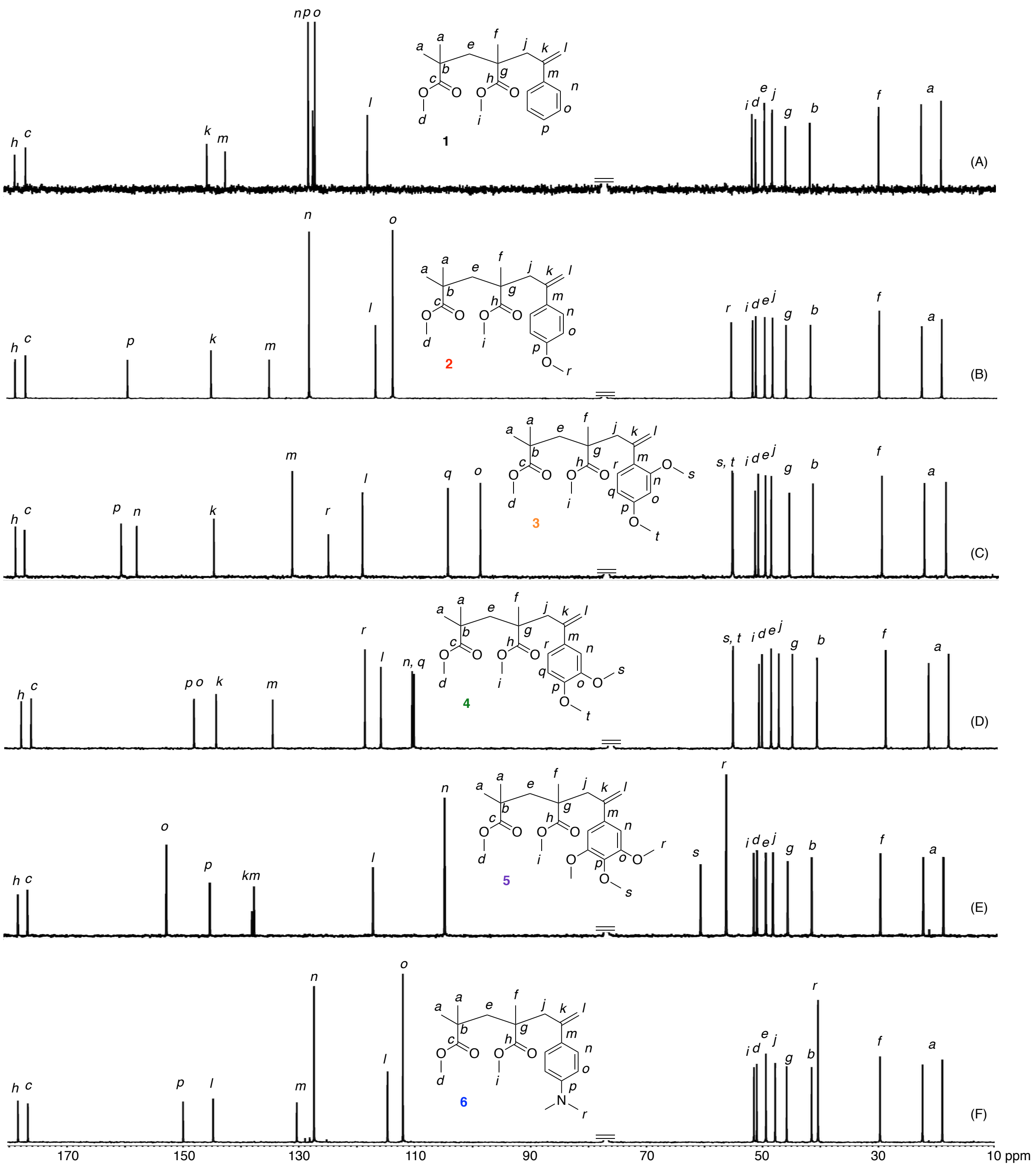

Figure S1. ^{13}C NMR spectra ($CDCl_3$, 55 °C) of sulfur-free RAFT agents (**1** (A), **2** (B), **3** (C), **4** (D), **5** (E) **6** (F)).

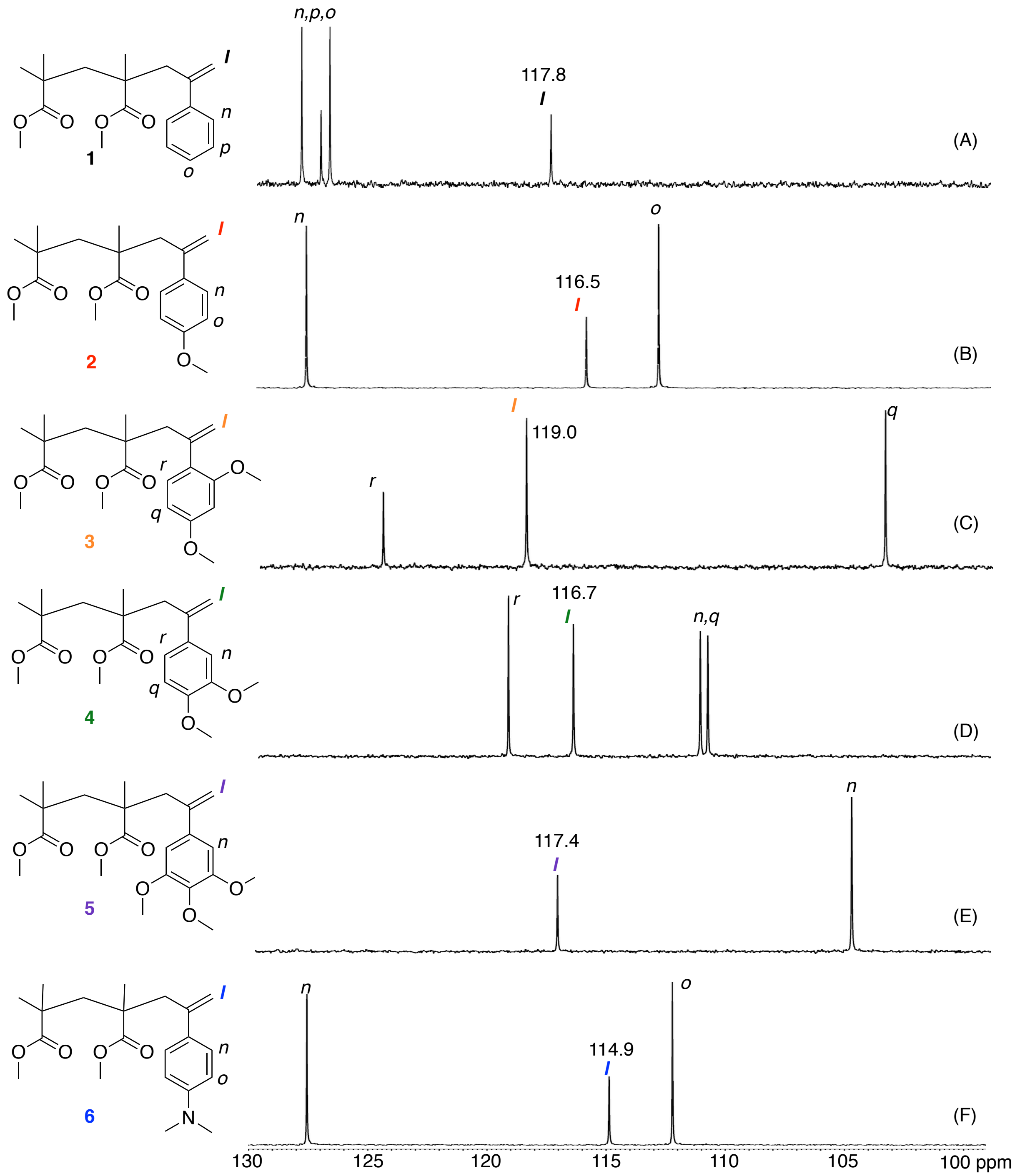

Figure S2. Expanded (100–130 ppm) ^{13}C NMR spectra ($CDCl_3$, 55 °C) of sulfur-free RAFT agents (**1** (A), **2** (B), **3** (C), **4** (D), **5** (E) **6** (F)).

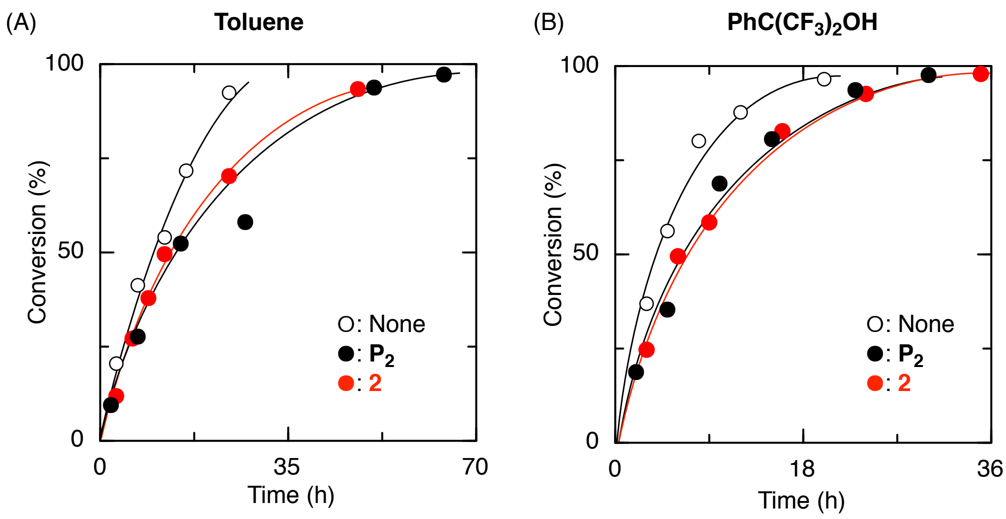

Figure S3. Time-conversion curves for radical polymerization of MMA in the absence and presence of $\mathbf{P_2}$ or **2** in toluene (A) or $PhC(CF_3)_2OH$ (B) at 60 °C: $[MMA]_0/[RAFT\ agent]_0/[AIBN]_0$ = 5000/0 or 50/5.0 mM.

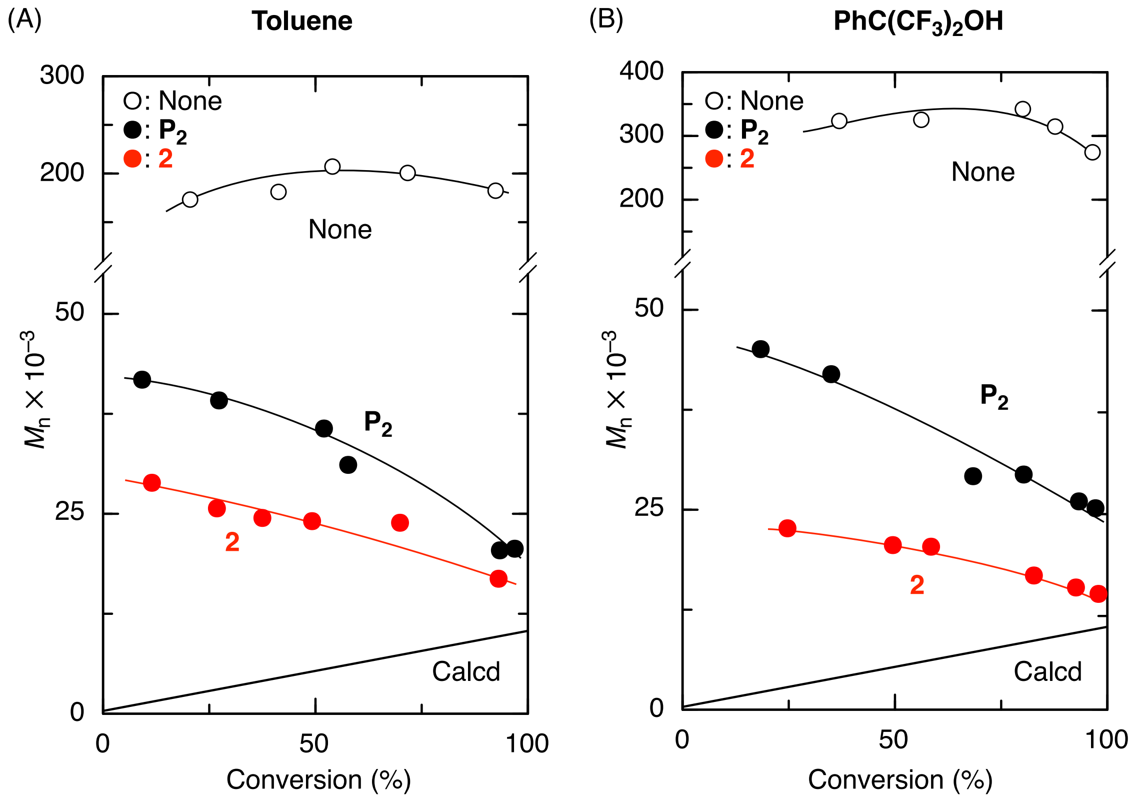

Figure S4. M_n values of polymers obtained in radical polymerization of MMA in the absence and presence of $\mathbf{P_2}$ or **2** in toluene (A) or $PhC(CF_3)_2OH$ (B) at 60 °C: $[MMA]_0/[RAFT\ agent]_0/[AIBN]_0$ = 5000/0 or 50/5.0 mM.

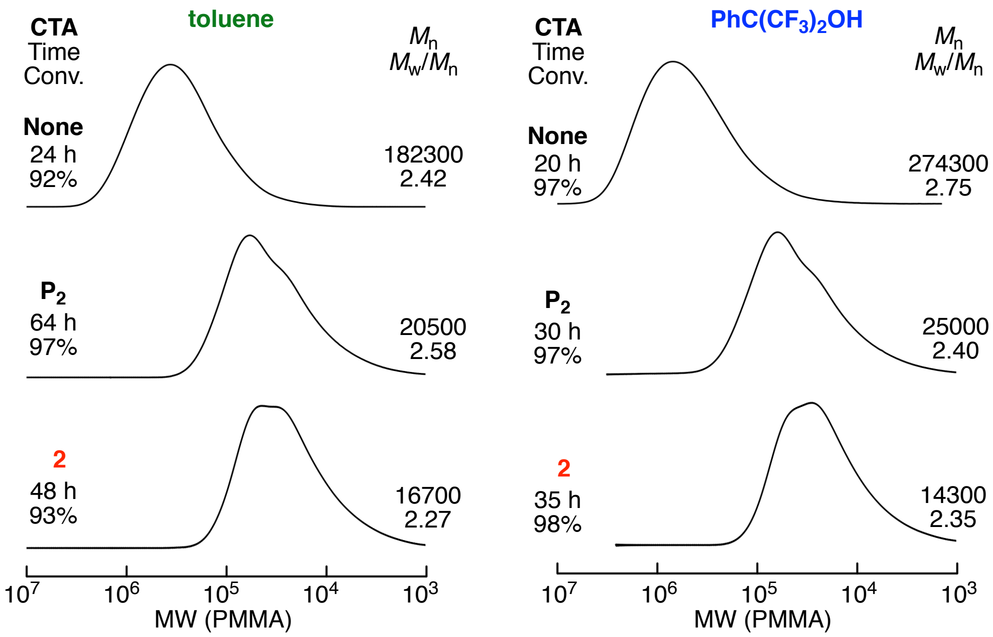

Figure S5. SEC curves of polymers obtained in radical polymerization of MMA in the absence and presence of $\mathbf{P_2}$ or **2** in toluene (A) or $PhC(CF_3)_2OH$ (B) at 60 °C: $[MMA]_0/[RAFT\ agent]_0/[AIBN]_0$ = 5000/0 or 50/5.0 mM.

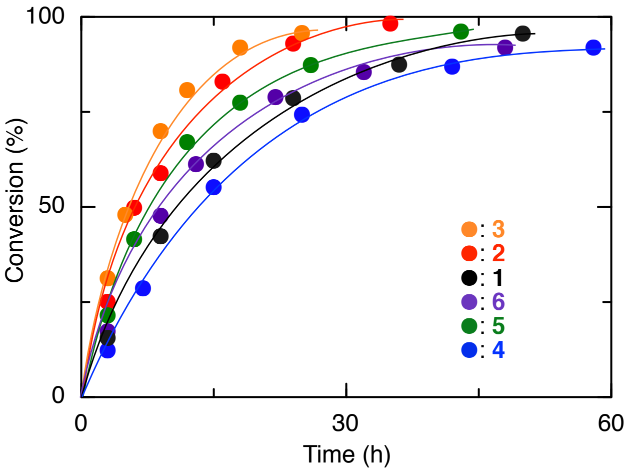

Figure S6. Time-conversion curves for radical polymerization of MMA in the presence of various sulfur-free RAFT agents in $PhC(CF_3)_2OH$ at 60 °C: $[MMA]_0/[RAFT\ agent]_0/[AIBN]_0$ = 5000/50/5.0 mM.

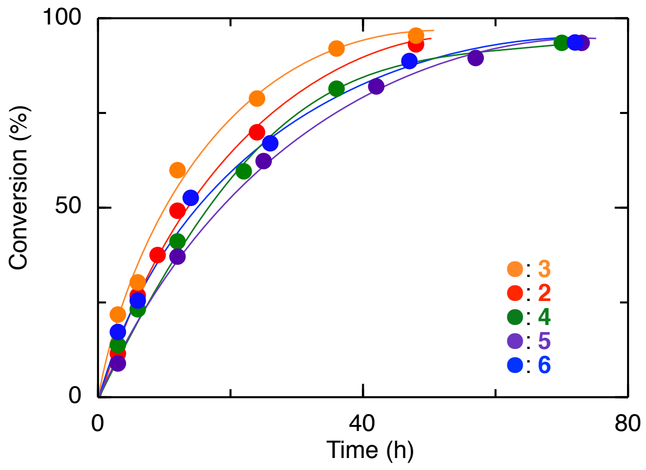

Figure S7. Time-conversion curves for radical polymerization of MMA in the presence of various sulfur-free RAFT agents in toluene at 60 °C: $[MMA]_0/[RAFT\ agent]_0/[AIBN]_0$ = 5000/50/5.0 mM.

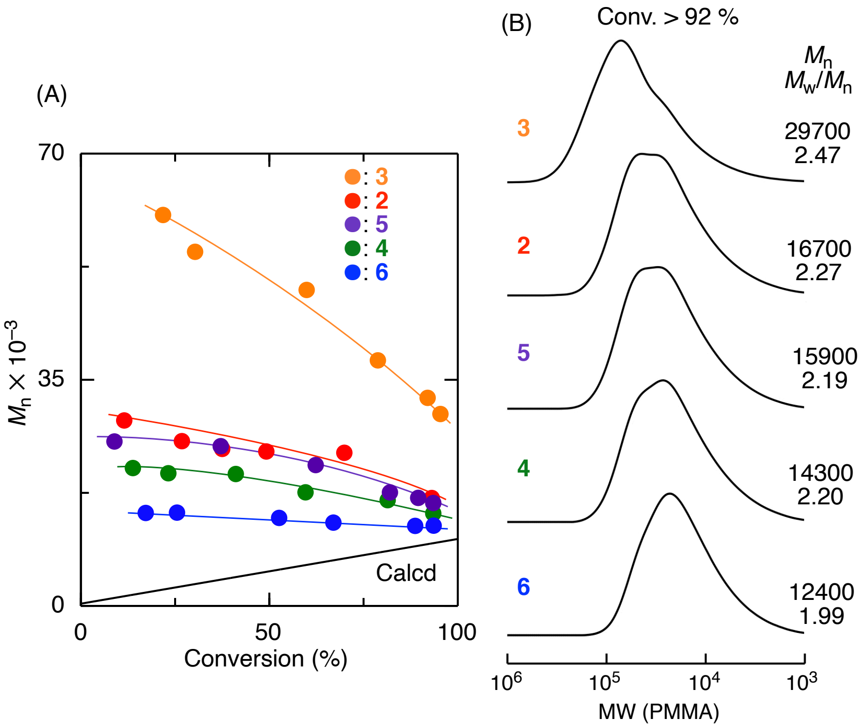

Figure S8. M_n values (A) and SEC curves (B) of PMMA obtained in radical polymerization of MMA in the presence of various sulfur-free RAFT agents in toluene at 60 °C: $[MMA]_0/[RAFT\ agent]_0/[AIBN]_0$ = 5000/50/5.0 mM.

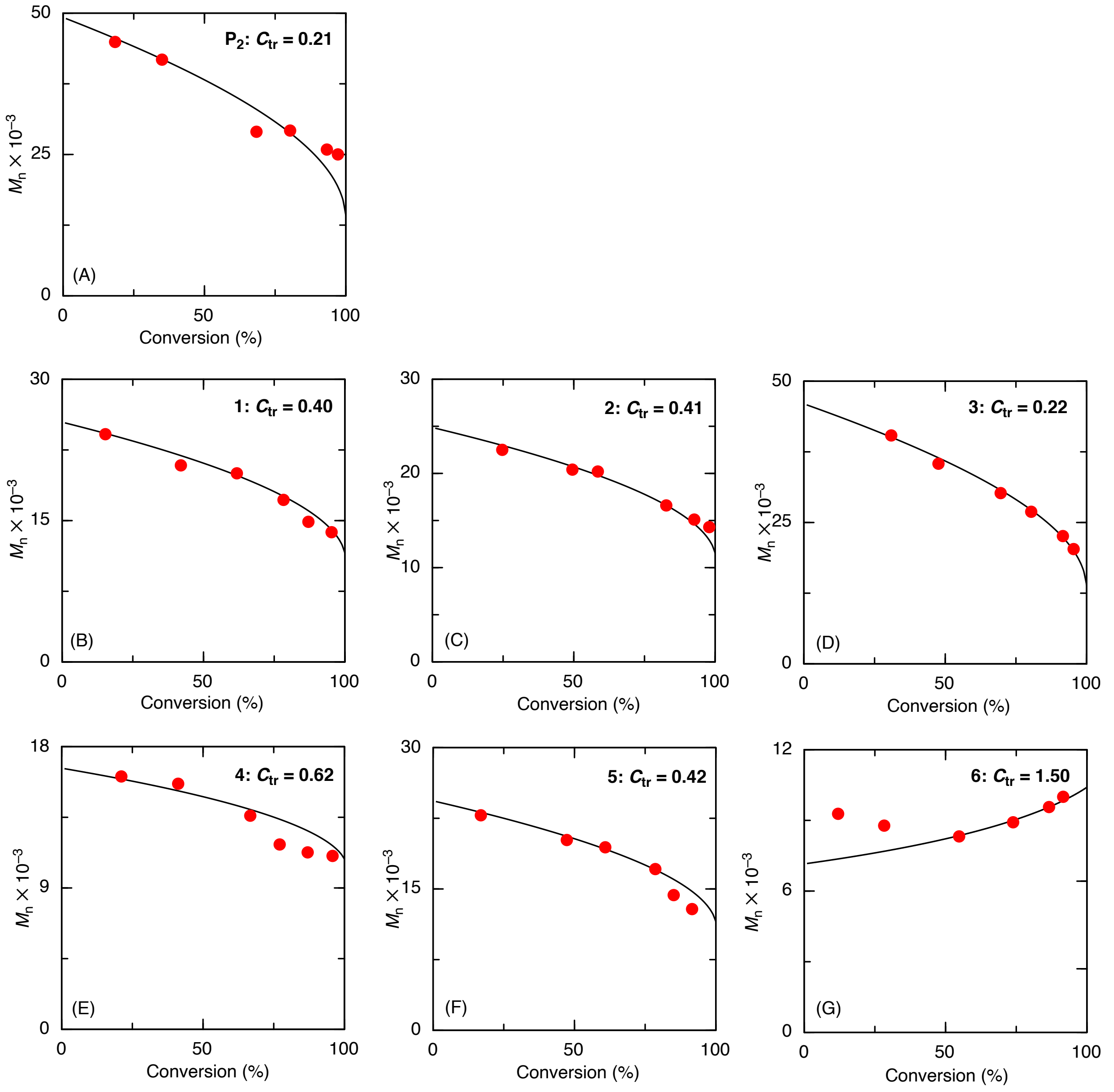

Figure S9. Estimation of chain-transfer constants (C_{tr}) for various sulfur-free RAFT agents using curve fitting for the M_n versus conversion plots based on the equation in the experimental section: $[MMA]_0/[RAFT\ agent]_0/[AIBN]_0$ = 5000/50/5.0 mM in $PhC(CF_3)_2OH$ at 60 °C.

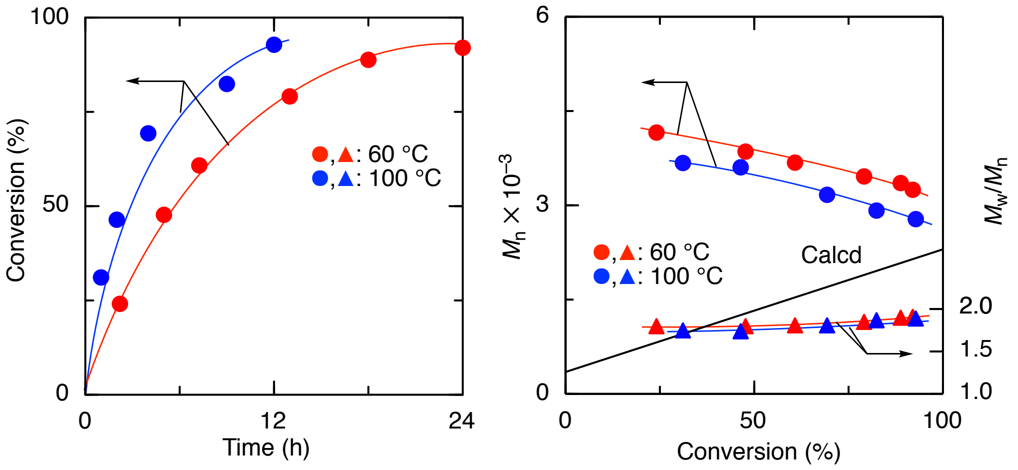

Figure S10. Effects of temperature on radical polymerization of MMA in the presence of **2** in PhC(CF_3)$_2$OH at 60 and 100 °C: [MMA]$_0$/[**2**]$_0$/[radical initiator]$_0$ = 1000/50/5.0 mM. Radical initiator: AIBN (60 °C) or VAm-110 (100 °C).

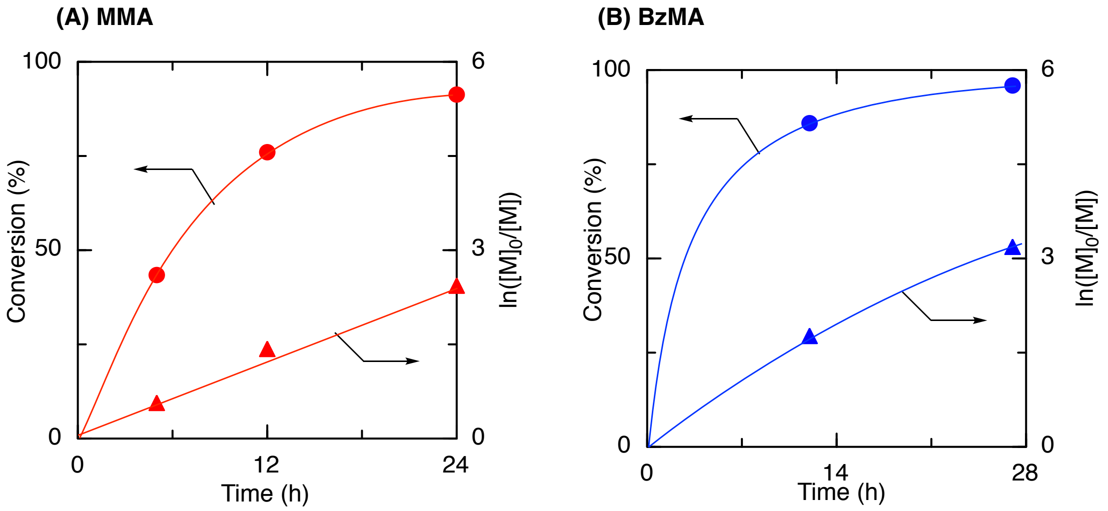

Figure S11. Time-conversion curves and first order kinetic plots for chain-extension reaction of MMA (A) and block copolymerization of BzMA (B) using PMMA obtained from **4** as a macro RAFT agent in PhC(CF_3)$_2$OH at 60 °C: [MMA of BzMA]$_0$/[macro RAFT agent]$_0$/[AIBN]$_0$ = 1000/50/5.0 mM.

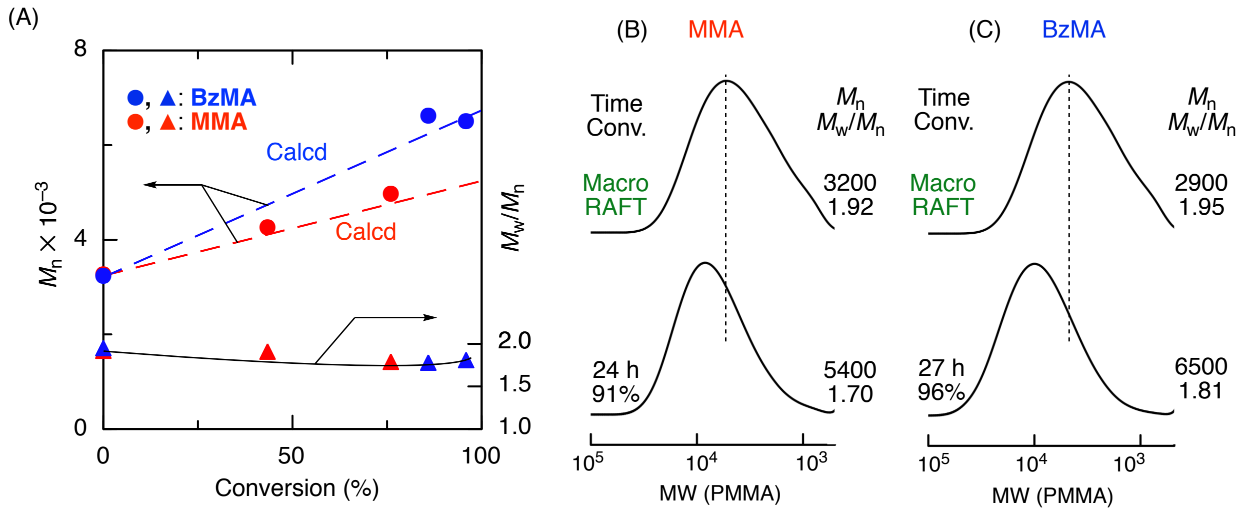

Figure S12. Chain-extension reaction of MMA (A, B) and block copolymerization of BzMA (A, C) using PMMA obtained from **4** as a macro RAFT agent in $PhC(CF_3)_2OH$ at 60 °C: [MMA or BzMA]$_0$/[macro RAFT agent]$_0$/[AIBN]$_0$ = 1000/50/5.0 mM.

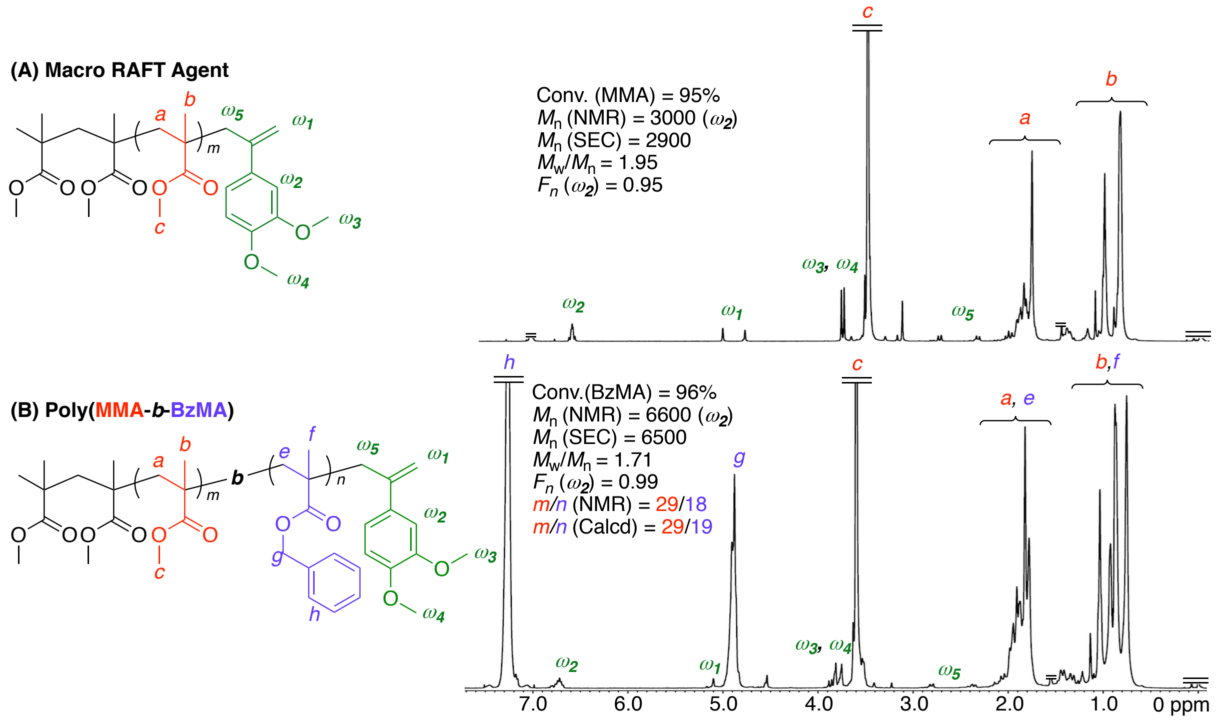

Figure S13. ^{1}H NMR spectra ($CDCl_3$, 55 °C) of PMMA as a macro RAFT agent obtained from **4** (A) and PMMA-*b*-PBzMA block copolymer obtained from the PMMA as a macro RAFT agent (B).

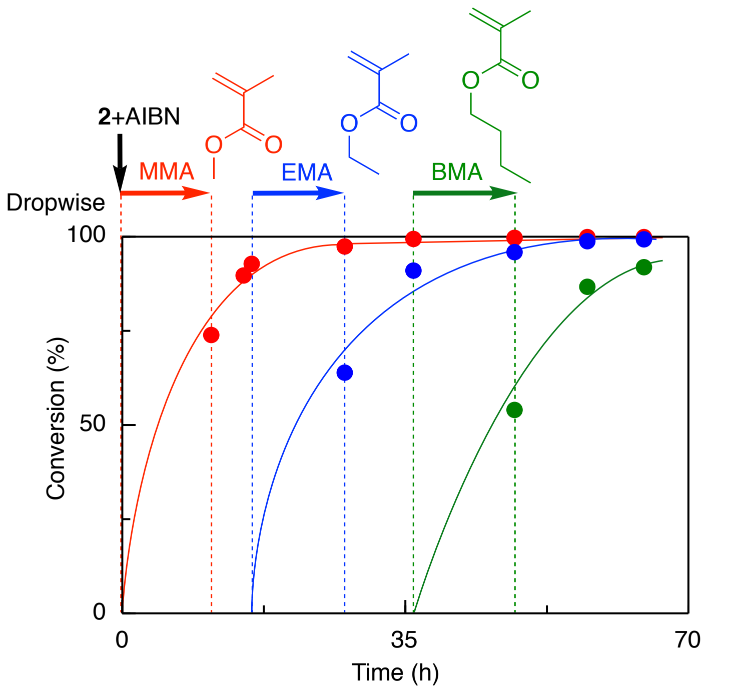

Figure S14. Time-conversion curves for the synthesis of triblock copolymer via continuous monomer addition using a syringe pump at 2.0 μL min^{-1} in $PhC(CF_3)_2OH$ at 60 °C: $[MMA]_0/[EMA]_{add}/[BMA]_{add}/[\mathbf{2}]_0/[AIBN]_0$ = 1000/1000/1000/50/20 mM.

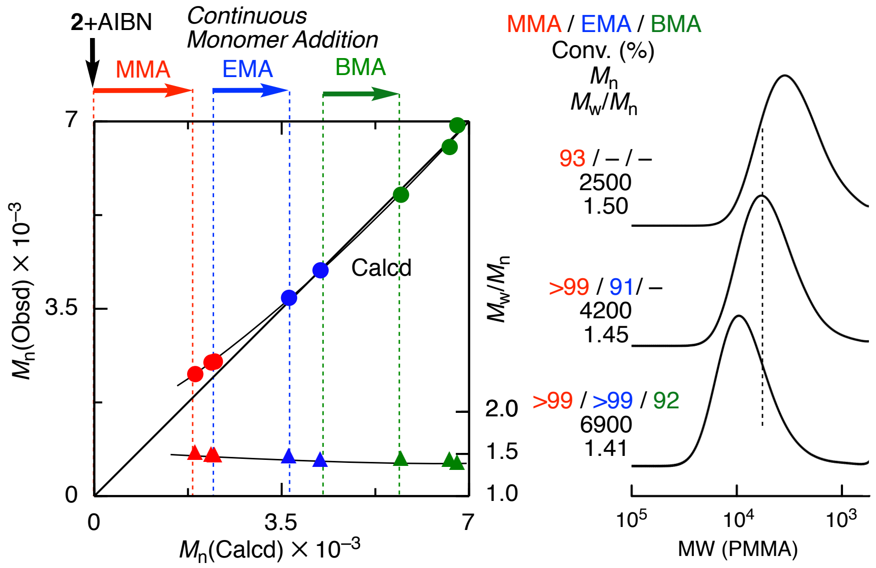

Figure S15. M_n values and SEC curves of polymers obtained via continuous monomer additions using a syringe pump at 2.0 μL min^{-1} in $PhC(CF_3)_2OH$ at 60 °C: $[MMA]_0/[EMA]_{add}/[BMA]_{add}/[\mathbf{2}]_0/[AIBN]_0$ = 1000/1000/1000/50/20 mM.

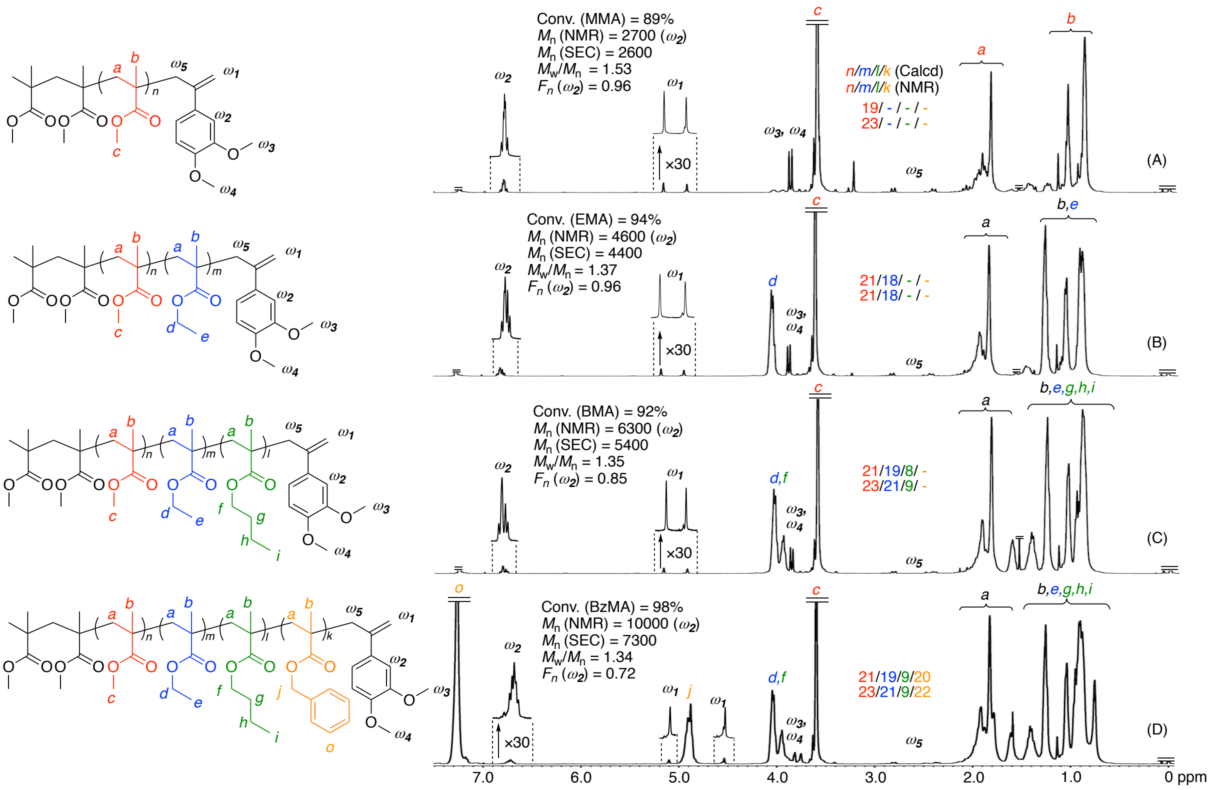

Figure S16. ^{1}H NMR spectra ($CDCl_3$, 55 °C) of PMMA (A), PMMA-*b*-PEMA diblock copolymer (B), PMMA-*b*-PEMA-PBMA triblock copolymer (C), and PMMA-*b*-PEMA-*b*-PBMA-*b*-PBzMA tetrablock copolymer (D) obtained in the same experiment as Figure 5.

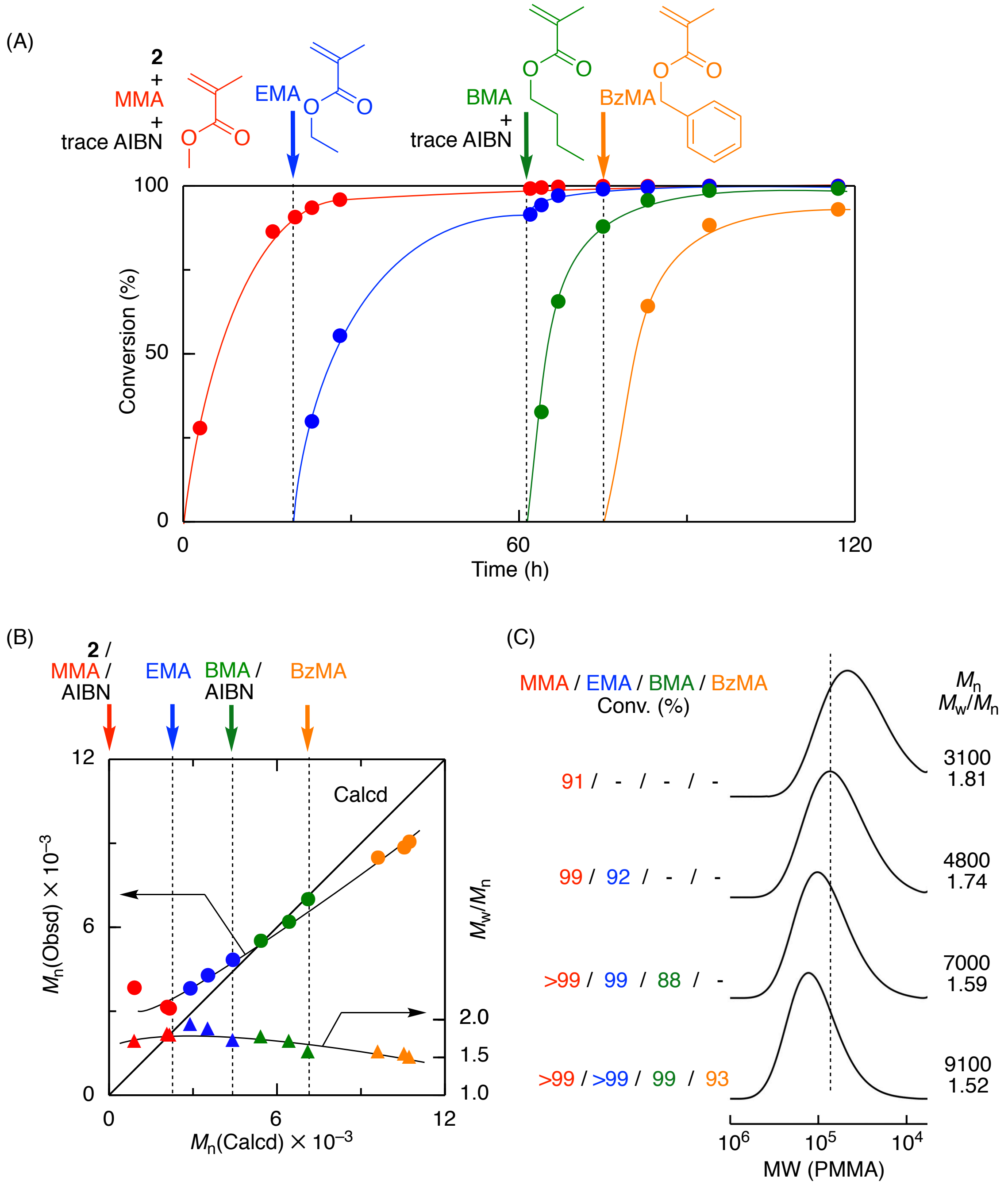

Figure S17. Time-conversion curves (A), M_n values (B), and SEC (C) curves of polymers obtained via monomer additions without using a syringe pump: $[MMA]_0/[EMA]_{add}/[BMA]_{add}/[BzMA]_{add}/[\mathbf{4}]_0$ = 1000/1000/1000/1000/50 mM in $PhC(CF_3)_2OH$ at 60 °C, $[AIBN]_{total} = [AIBN]_0 + [AIBN]_{add}$ = 5.0 + 5.0 mM.

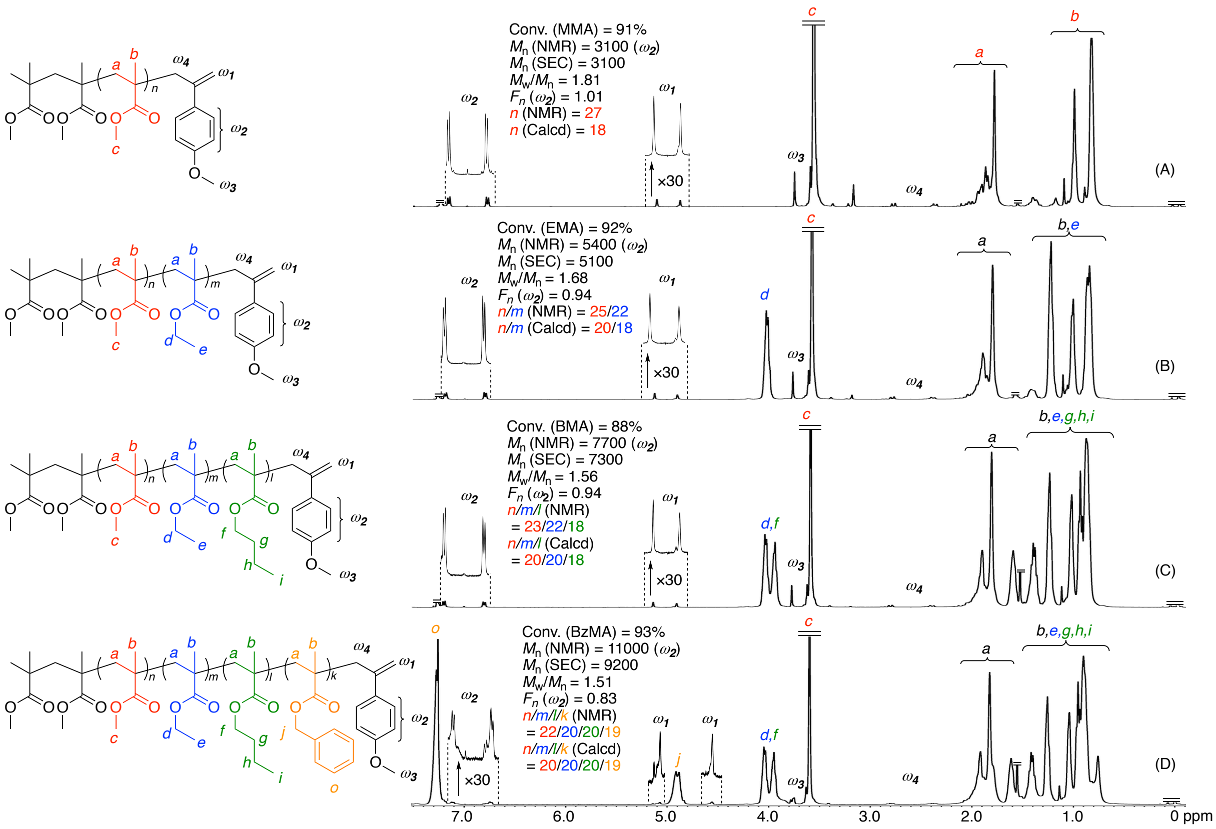

Figure S18. ^{1}H NMR spectra ($CDCl_3$, 55 °C) of PMMA (A), PMMA-*b*-PEMA diblock copolymer (B), PMMA-*b*-PEMA-*b*-PBMA triblock copolymer (C), and PMMA-*b*-PEMA-*b*-PBMA-*b*-PBzMA tetrablock copolymer (D) obtained in the same experiment as Figure S17.
